# Chemical Strategies for Reducing Polymerization Shrinkage Stress in Experimental Dental Resin-Based Materials: A Scoping Review

**DOI:** 10.3390/dj14080479

**Published:** 2026-08-05

**Authors:** Ionuț Tărăboanță, Nicoleta Ilie, Andra Claudia Tărăboanța-Gamen, Gianina Iovan, Simona Stoleriu, Sorin Andrian

**Affiliations:** 1 Grigore T. Popa University of Medicine and Pharmacy, 16 Universitatii Str., 700115 Iasi, Romania; ionut-taraboanta@umfiasi.ro (I.T.); gianina.iovan@umfiasi.ro (G.I.); simona.stoleriu@umfiasi.ro (S.S.); sorin.andrian@umfiasi.ro (S.A.); 2 Department of Conservative Dentistry, University Hospital, Ludwig-Maximilians-University, Goethestr. 70, D-80336 Munich, Germany; nicoleta.ilie@med.uni-muenchen.de

**Keywords:** chemical strategies, dental composites, experimental monomers, polymerization shrinkage stress, resin-based materials, scoping review

## Abstract

**Background**: Polymerization shrinkage stress remains one of the main limitations of methacrylate-based dental resin composites, as it may lead to marginal gap formation, interfacial debonding, and long-term restoration failure. In recent years, numerous experimental monomers and alternative polymerization strategies have been proposed to mitigate stress development during polymer network formation. This scoping review aimed to map and summarize experimental and modified monomer systems investigated for reducing polymerization shrinkage stress in dental methacrylate-based composites. **Methods**: A comprehensive literature search was conducted in PubMed/MEDLINE, Scopus, Web of Science Core Collection, Embase, and Google Scholar to identify relevant studies published between 2010 and 2025. The search strategy combined terms related to dental resins, polymerization shrinkage, shrinkage stress, and experimental monomers (1678 papers found). After duplicate removal and screening of titles and abstracts, potentially relevant articles were assessed for full-text eligibility according to predefined inclusion criteria. In vitro studies investigating experimental monomers or modified resin systems with reported polymerization shrinkage stress measurements were included. Data extraction focused on monomer composition, chemical strategy for stress reduction, measurement methods, and reported shrinkage stress values. **Results**: A total of 33 studies met the eligibility criteria and were included in the qualitative synthesis. The reviewed studies investigated a wide range of molecular strategies, including thiol–ene and thiourethane chemistries, addition–fragmentation chain transfer (AFCT) networks, ring-opening or expanding monomers, high-molecular-weight dimethacrylates, ether-based monomers, and alternative reactive diluents. Reported polymerization shrinkage stress outcomes varied widely across studies because of differences in testing methods, specimen geometry, system compliance, curing protocols, reporting units, and control materials. Therefore, the results were synthesized descriptively, with emphasis on within-study comparisons between experimental systems and their respective controls rather than on direct numerical comparisons across studies. Thiol-based systems and adaptive polymerization mechanisms consistently demonstrated the greatest reductions in shrinkage stress compared with conventional dimethacrylate resin matrices. **Conclusions**: Experimental monomer design represents a promising strategy for controlling polymerization shrinkage stress in dental composites. Chemical approaches that modify polymerization mechanisms or network architecture may significantly reduce stress development during curing. Further studies are required to evaluate the long-term chemical, physical, and mechanical stability, as well as the clinical applicability, of these experimental and modified resin systems.

## 1. Introduction

Free-radical polymerization of methacrylate-based resins underpins the vast majority of contemporary restorative dental materials, including adhesive systems, resin-based composites, and resin cements [1]. These materials commonly rely on dimethacrylate monomers such as bisphenol A-glycidyl methacrylate (Bis-GMA), urethane dimethacrylate (UDMA), and triethylene glycol dimethacrylate (TEGDMA), which form highly cross-linked polymer networks upon light-activated polymerization. Despite their widespread clinical use and favorable mechanical properties, methacrylate-based systems are intrinsically associated with volumetric contraction during polymer network formation. This dimensional change results from the conversion of monomeric species into densely crosslinked structures and generates internal stresses within the material and at the bonded interface with dental tissues [2,3]. The stresses generated during this process, commonly referred to as polymerization shrinkage stress, are widely recognized as a major factor contributing to marginal gap formation, interfacial debonding, microcrack development, and bacterial infiltration at the tooth–restoration interface. These phenomena may ultimately compromise the longevity of restorations by facilitating secondary caries and degradation of the adhesive interface [4].

Polymerization shrinkage, polymerization shrinkage stress, elastic modulus development, and degree of conversion represent related but distinct outcomes. Shrinkage describes volumetric contraction during network formation, whereas shrinkage stress reflects the stress generated when this contraction is constrained. Therefore, stress development depends not only on shrinkage magnitude but also on gelation, stress relaxation, elastic modulus development, degree of conversion, cavity configuration, and testing system compliance [2,3].

Historically, strategies aimed to reduce shrinkage-related effects have relied primarily on compositional modifications of conventional methacrylate formulations. These approaches include adjusting the ratios of commonly used dimethacrylates such as Bis-GMA, UDMA, and TEGDMA, as well as increasing the inorganic filler content of resin-based composites [5]. Although such strategies may partially reduce overall polymerization shrinkage, they are associated with inherent limitations. Increasing resin viscosity or reducing the concentration of low-molecular-weight diluent monomers can decrease shrinkage but may simultaneously compromise the degree of conversion, handling properties, or the development of optimal mechanical properties. As a result, research efforts over the past decade have increasingly focused on the rational design of experimental monomers and alternative polymerization chemistry to modify the polymerization process itself [6,7,8].

Several innovative chemical strategies have emerged from this research direction. Step-growth polymerization systems such as thiol–ene and thiol–yne networks have been shown to delay gelation and reduce stress accumulation during polymer network formation. Similarly, thiourethane oligomers can promote chain-transfer reactions that delay the gel point and facilitate stress relaxation during curing. Other strategies include addition–fragmentation chain transfer (AFCT) mechanisms, which enable reversible bond cleavage and dynamic rearrangement of the polymer network, thereby allowing the developing material to accommodate shrinkage-induced stresses [9]. Additional approaches involve the use of ring-opening or expanding monomers, high-molecular-weight dimethacrylates designed to reduce reactive group density, bio-based monomers, ether-based vinyl systems, and alternative reactive diluents intended to replace conventional TEGDMA. Although chemically diverse, these strategies share a common objective: decreasing the polymerization-induced stress while maintaining adequate conversion and mechanical properties of the final composite material [10,11,12].

Parallel with the development of these novel resin systems, a variety of experimental techniques have been used to quantify polymerization shrinkage stress. Commonly used methods include tensometers, cantilever beam systems such as the Bioman device, universal testing machines configured for stress analysis, and other specialized polymerization stress analyzers [13,14]. However, variations in experimental setups, specimen geometry, curing protocols, and reporting units often complicate the direct comparison of the results across different studies. Consequently, synthesizing the available evidence requires a structured approach that considers both the chemical strategies employed and the experimental methodologies used to evaluate shrinkage stress.

Given the increasing diversity of experimental monomer systems and polymerization mechanisms proposed in recent years, a comprehensive overview of the available evidence is necessary to clarify current research trends and identify the most promising strategies for reducing polymerization shrinkage stress in dental resin systems. Such an analysis may also contribute to a better understanding of how molecular design and polymerization kinetics influence stress development during curing.

Accordingly, the present scoping review aims to systematically map and synthesize the scientific literature published between 2010 and 2025 concerning experimental monomers and polymer systems developed specifically to reduce polymerization shrinkage stress in resin-based dental composites. Particular emphasis is placed on identifying the principal chemical strategies investigated, the types of experimental monomers employed, the experimental methods used to measure shrinkage stress, and the magnitude of stress reduction reported in comparison with conventional dimethacrylate resin systems [15,16].

## 2. Materials and Methods

The review methodology followed the recommendations of the PRISMA Extension for Scoping Reviews (PRISMA-ScR) to ensure transparency and reproducibility in the identification, selection, and synthesis of the available evidence [17,18].

The objective of the methodological framework was to identify studies investigating novel monomers, co-monomers, or polymerization mechanisms specifically designed to reduce polymerization shrinkage stress in resin-based dental materials. Particular emphasis was placed on chemical strategies that modify the polymerization process or network architecture, such as addition–fragmentation chain transfer (AFCT), thiol-ene and thiourethane chemistries, ring-opening or expanding monomers, high-molecular-weight dimethacrylates, and alternative diluent monomers.

The study selection process consisted of a structured multi-stage approach, including database search, duplicate removal, title and abstract screening, full-text eligibility assessment, and data extraction. All stages were conducted independently by two reviewers, and any disagreements were resolved through discussion and consensus.

The overall study identification and screening workflow was documented using a PRISMA-ScR flow diagram illustrating the number of records identified, screened, excluded, and included in the final synthesis.

### 2.1. Research Questions

The present scoping review was guided by the following research questions:

What experimental monomers or resin systems have been investigated between 2010 and 2025 to reduce polymerization shrinkage stress in resin-based dental composites?

What chemical strategies have been proposed to mitigate polymerization shrinkage stress in these systems?

What experimental methods are used to measure polymerization shrinkage stress in dental resin systems?

To what extent do experimental monomer systems reduce polymerization shrinkage stress compared with conventional dimethacrylate matrices such as Bis-GMA/TEGDMA?

These research questions were designed to map the current state of research regarding chemical strategies aimed at reducing polymerization shrinkage stress in dental composite materials.

### 2.2. Information Sources and Search Strategy

A comprehensive literature search was conducted to identify studies investigating experimental monomers and resin systems developed to reduce polymerization shrinkage stress in dental resin-based materials. The search strategy was designed to capture experimental formulations and novel polymer systems evaluated for their ability to reduce stress generation during polymerization. To ensure broad coverage of the available evidence, multiple electronic databases widely used in biomedical and materials science research were consulted.

The electronic search was performed in PubMed/MEDLINE, Scopus, Web of Science Core Collection, Embase, and Google Scholar. Google Scholar was used only as a supplementary source to identify potentially relevant records not retrieved from the main bibliographic databases. The primary Google Scholar query was “dental composite” “polymerization shrinkage stress” “experimental monomer”. Additional targeted searches were performed using chemistry-specific combinations, including “dental composite” “shrinkage stress” thiourethane, “dental composite” “shrinkage stress” “thiol-ene”, “dental composite” “shrinkage stress” AFCT, “dental composite” “shrinkage stress” “ring-opening”, and “dental composite” “shrinkage stress” nanogel. Results were screened in the default relevance-based order, and the first 200 records were assessed manually.

The publication period is clarified in Section 2. Eligible studies were those published between 1 January 2010 and 31 December 2025. The final electronic search was performed on 27 January 2026 to capture studies published or indexed toward the end of 2025. We also clarified that online-ahead-of-print articles were eligible only when the full text was available and the publication or online publication date fell within the predefined period. Records published or indexed after 31 December 2025 were not included. Late-2025 publications were identified through the final database searches, Google Scholar screening, and citation tracking.

**Figure 1:**
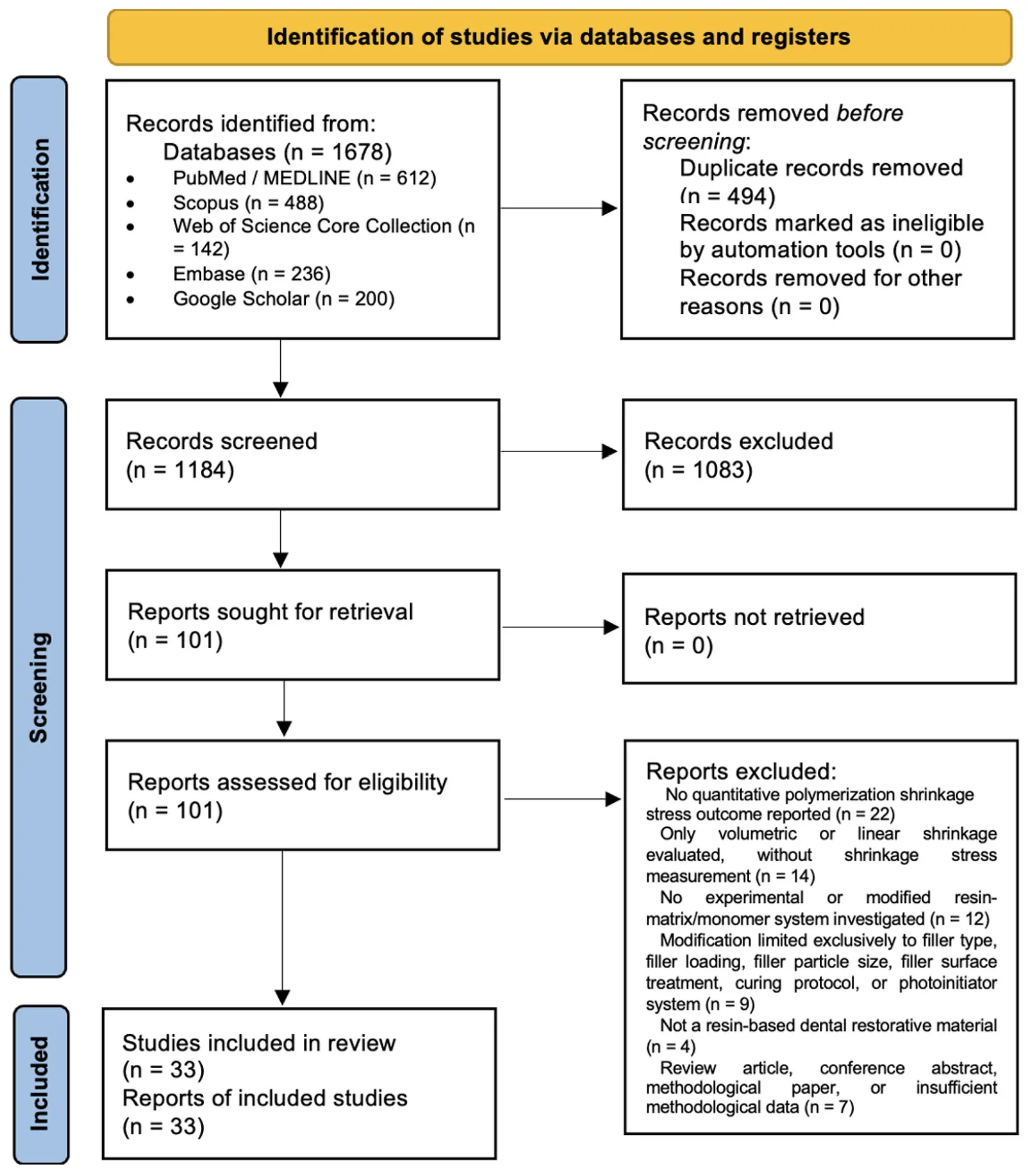
PRISMA-ScR study flow diagram.

The search strategy was designed to capture both general and chemistry-specific approaches for reducing polymerization shrinkage stress in resin-based dental restorative materials. Search terms combined concepts related to dental composites, resin-based materials, polymerization shrinkage stress, contraction stress, and experimental or modified monomer systems. Chemistry-specific terms included thiourethane, thiol-ene, thiol-yne, addition–fragmentation chain transfer, AFCT, allyl sulfide, trithiocarbonate, ring-opening polymerization, spiro-orthocarbonate, silorane, oxirane, vinylcyclopropane, nanogel, low-shrinkage resin, and stress-relieving monomer. The complete search strategy used for each database is provided in Supplementary Table S1, and the study identification and selection process is summarized in the PRISMA-style flow diagram presented in Figure 1.

An example of the search string applied in PubMed/MEDLINE is as follows:

(“dental resin*” OR “dental composite*” OR “restorative resin*” OR “adhesive system”) AND (“polymerization shrinkage” OR “curing contraction” OR “shrinkage stress”) AND (“experimental monomer” OR “novel monomer” OR “alternative co-monomer” OR “polymer network modification”).

Search syntax was adapted to the indexing structure and functionality of each database. In addition, the reference lists of all articles selected for full-text analysis were manually screened to identify additional relevant studies that may not have been retrieved during the electronic search.

All records retrieved from the databases were exported to Mendeley reference management software, version 1.19.8 (Elsevier, Amsterdam, The Netherlands), which was used to identify and remove duplicate entries prior to the screening process.

The detailed electronic search strategy and the number of records identified in each database are presented in Table 1.

Two reviewers independently screened the titles and abstracts of all retrieved records according to the predefined eligibility criteria. Full-text articles were then assessed independently by the same reviewers. Inter-reviewer agreement was calculated using Cohen’s kappa coefficient. The observed agreement was 0.942, the agreement expected by chance was 0.597, and Cohen’s kappa was 0.86, indicating excellent agreement. Disagreements were resolved through discussion until a consensus was reached.

### 2.3. Eligibility Criteria

Predefined eligibility criteria were established to ensure the relevance and methodological consistency of the studies included in this scoping review. These criteria were applied during both the title and abstract screening phase and the full-text assessment stage.

Studies were considered eligible if they met the following inclusion criteria: (1) in vitro experimental studies investigating dental resin-based materials; (2) studies reporting the development or incorporation of experimental monomers, co-monomers, or modified resin matrices intended to influence polymerization behavior; (3) investigations evaluating polymerization shrinkage stress as a primary or secondary outcome; (4) articles published between 2010 and 2025; and (5) studies with full text available in English.

Studies were excluded if they met any of the following exclusion criteria: (1) investigations evaluating only commercial resin-based materials without modifications to the monomer composition; (2) studies reporting exclusively volumetric or linear shrinkage measurements without quantitative assessment of shrinkage stress; (3) research focusing primarily on filler modifications, curing protocols, or photoinitiator systems without introducing novel monomeric components; (4) publications lacking sufficient methodological detail regarding the experimental formulation; and (5) review articles, editorials, conference abstracts, or methodological papers without original experimental data.

Conference papers retrieved through database filters were screened during the selection process; however, conference abstracts or proceedings papers without sufficient methodological details or without full peer-reviewed experimental data were excluded.

Any discrepancies regarding study eligibility were resolved through discussion and consensus. Only studies meeting all inclusion criteria and none of the exclusion criteria were retained for qualitative synthesis.

The included studies were not segregated according to regular incremental-fill, bulk-fill, paste-like, or flowable clinical categories because the primary focus of this scoping review was the resin matrix chemistry and monomeric strategy used to reduce polymerization shrinkage stress. When such formulation categories were reported in the original studies, they were considered as background material characteristics rather than as primary comparison variables.

The inclusion and exclusion criteria applied during title/abstract screening and full-text assessment are summarized in the study selection flow diagram presented in Figure 1.

### 2.4. Data Extraction and Organization Process

Data extraction was performed using a standardized data collection form developed prior to the analysis in order to ensure consistency and reproducibility of the extracted information. Each study that met the inclusion criteria was independently reviewed by two investigators, who extracted and recorded the relevant methodological and experimental data.

The extracted information included the first author and year of publication, the type of dental material investigated, and the experimental monomers or resin systems incorporated into the formulation. Particular attention was given to identifying the chemical strategy employed to reduce polymerization shrinkage stress, such as the incorporation of high-molecular-weight monomers, urethane-based systems, ring-opening monomers, adaptive stress-relaxation mechanisms, or alternative polymerization chemistries.

In addition, quantitative data related to polymerization shrinkage stress outcomes were extracted whenever reported. The measurement methods used to determine shrinkage stress, including cantilever beam systems, tensometers, universal testing machines, or other experimental setups, were also recorded. When available, the numerical values of polymerization shrinkage stress were documented to facilitate comparison between experimental formulations and conventional dimethacrylate reference systems.

All extracted data were subsequently organized into a structured synthesis table to enable comparison across studies and identify overarching research trends in experimental monomer development. The table included the following variables: author and year, dental material type, experimental monomer or resin system, chemical strategy for shrinkage stress reduction, shrinkage outcome evaluated, measurement method, reported shrinkage stress values, and the main conclusions related to polymerization shrinkage stress.

When information was not clearly described in the main manuscript, additional details were obtained by consulting the Supplementary Materials or related publications from the same research group when available. Any discrepancies between reviewers during the data extraction process were resolved through discussion and consensus to ensure the accuracy of the final dataset. The data extraction template applied in the present review is presented in Table 2.

Polymerization shrinkage stress values were extracted and reported as presented in the original studies. Because the included studies used different measurement devices, specimen geometries, compliance settings, curing protocols, time points, reporting units, and control materials, stress values were not converted, pooled, or directly compared across studies. Numerical comparisons were interpreted only within the context of each individual study and its respective control group.

### 2.5. Methodological Reporting Quality Assessment

Although a formal risk-of-bias assessment is not a mandatory component of scoping reviews, a descriptive methodological reporting quality assessment was conducted to provide context for the interpretation of the reported findings [19]. The term “methodological reporting quality” was used instead of “risk of bias” because the included studies were in vitro laboratory-based investigations of dental resin systems rather than clinical intervention studies.

The assessment focused on key methodological domains considered relevant for in vitro investigations of polymerization shrinkage stress in resin-based dental materials: (1) clarity of resin formulation and specimen preparation; (2) reporting of photopolymerization parameters, including light source characteristics, irradiance, and exposure time; (3) suitability and description of the shrinkage stress measurement method; (4) completeness and transparency of outcome reporting, including units and time points; and (5) adequacy of the statistical analysis.

Each domain was categorized as presenting Low, Unclear, or High concern. A Low concern rating was assigned when the relevant methodological information was clearly reported. An Unclear concern rating was assigned when reporting was incomplete but still allowed interpretation of the findings. A High concern rating was assigned when critical methodological information was missing or when the experimental design raised major concerns regarding the interpretation of the reported shrinkage stress values.

The overall judgment was derived from the individual domain ratings. An overall Low concern rating was assigned only when all or nearly all key domains were adequately reported and no domain was rated as High concern. An overall Unclear concern rating was assigned when one or more important domains were insufficiently reported but no major methodological concern was identified. An overall High concern rating was assigned when at least one critical domain was rated as High concern or when missing information substantially limited interpretation of the study findings.

The methodological reporting quality assessment was performed independently by two reviewers, and any disagreements were resolved through discussion and consensus. This assessment was conducted for descriptive purposes only and was not used as a criterion for study exclusion or weighting the included studies during data synthesis. Studies were excluded only when they did not meet the predefined eligibility criteria, did not report polymerization shrinkage stress outcomes, or provided insufficient methodological information to allow data extraction. The results of the assessment are summarized in Table 3.

## 3. Results

### 3.1. Study Selection

The literature search identified a total of 1678 records across all electronic databases. Specifically, 612 records were retrieved from PubMed/MEDLINE, 488 from Scopus, 142 from Web of Science Core Collection, 236 from Embase, and 200 records from Google Scholar, where the first 200 most relevant results were screened due to the large number of records retrieved.

After removing 494 duplicate records, 1184 unique articles remained for title and abstract screening. During this stage, 1083 records were excluded because they did not meet the inclusion criteria. The most common reasons for exclusion included studies unrelated to dental resin-based materials, investigations focusing exclusively on commercial products, studies addressing filler modifications without introducing novel monomer systems, and publications that did not evaluate polymerization shrinkage stress.

Following this initial screening, 101 articles were considered potentially relevant and were retrieved for full-text assessment. After applying the predefined eligibility criteria, 68 studies were excluded. The main reasons for exclusion included the absence of quantitative shrinkage stress data, investigations focusing exclusively on volumetric shrinkage, studies lacking experimental monomer development, and review or methodological papers without original experimental data.

Ultimately, 33 studies met all eligibility criteria and were included in the qualitative synthesis of this scoping review. The study selection process is summarized in the PRISMA-ScR flow diagram (Figure 1).

### 3.2. General Characteristics of the Included Studies

The 33 studies included in this review were published between 2010 and 2025 and investigated a wide range of experimental monomers and resin systems designed to reduce polymerization shrinkage stress in dental materials.

All included studies reported in vitro experimental investigations evaluating experimental resin formulations containing newly developed monomers or modified polymer networks. The investigated materials consisted primarily of resin-based dental composites, although several studies also examined unfilled experimental resins or adhesive formulations designed to assess the intrinsic polymerization behavior of newly synthesized monomers.

In most studies, the experimental formulations were compared with conventional dimethacrylate-based reference systems, typically based on bisphenol A-glycidyl methacrylate (Bis-GMA), urethane dimethacrylate (UDMA), and triethylene glycol dimethacrylate (TEGDMA) matrices. This comparative approach allowed the authors to evaluate the effectiveness of the proposed chemical strategies in reducing polymerization shrinkage stress relative to widely used dental resin systems.

The included studies demonstrated substantial diversity in both monomer design strategies and experimental methodologies. Experimental formulations included newly synthesized urethane-based monomers, high-molecular-weight dimethacrylates, ring-opening monomers, vinyl-based polymer systems, adaptive stress-relief additives, and multifunctional resin architectures. Depending on the formulation strategy, these components were incorporated either as primary matrix monomers or as functional additives within conventional resin systems.

### 3.3. Distribution of Studies by Year of Publication

**Figure 2:**
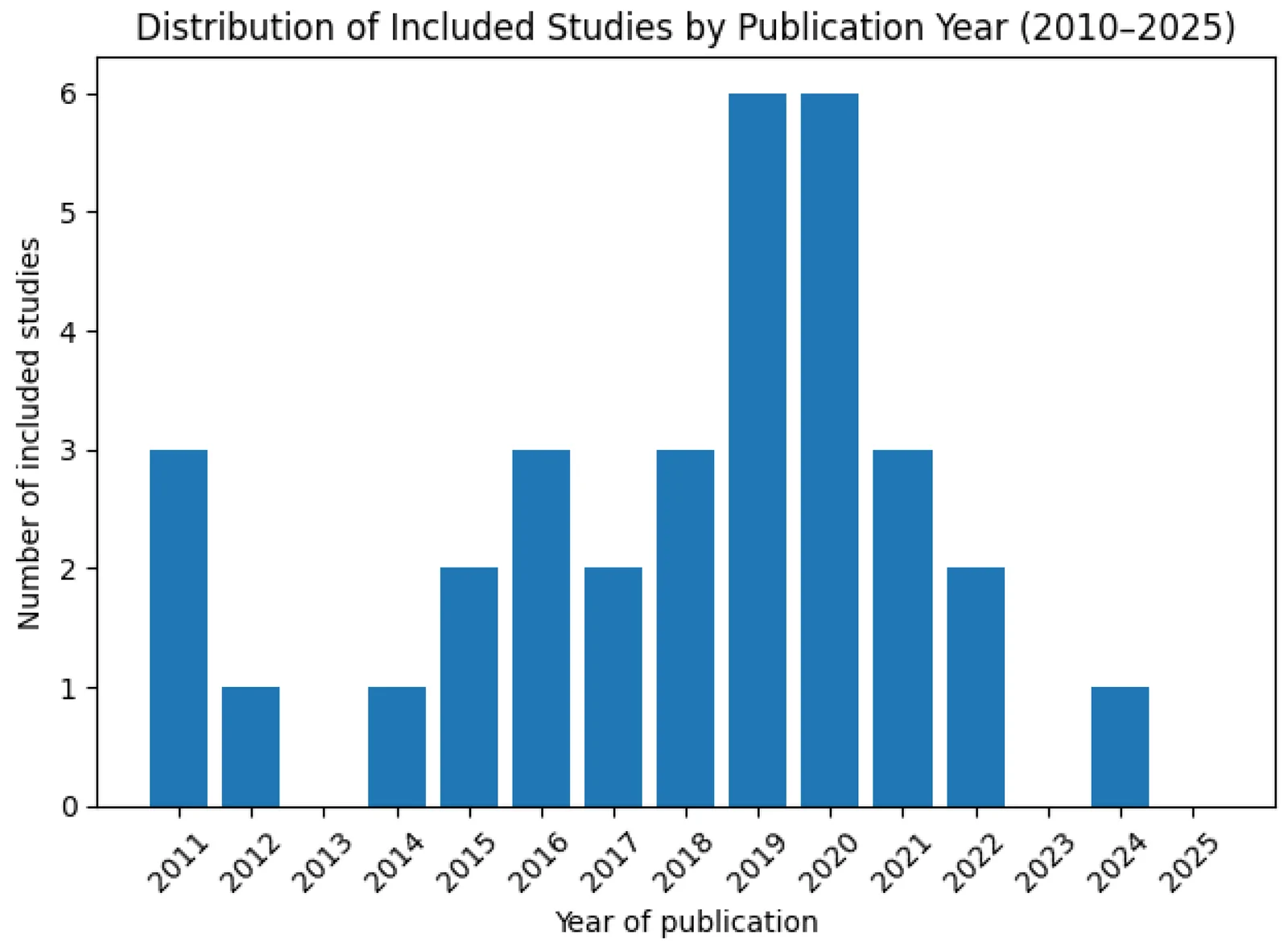
Distribution of included studies by publication year (2010–2025).

The temporal distribution of the included studies revealed a gradual increase in research activity related to experimental monomers designed to reduce polymerization shrinkage stress (Figure 2).

Early investigations published between 2010 and 2015 primarily focused on modified dimethacrylate systems and alternative monomers intended to replace or complement Bis-GMA-based matrices. These studies largely explored strategies aimed at reducing reactive group density or modifying polymer network architecture.

From 2016 onward, a broader diversification of chemical approaches was observed, including the development of urethane-derived oligomers, ring-opening monomer systems, and adaptive network modifiers capable of facilitating stress relaxation during polymerization.

A notable increase in research output was observed after 2020, coinciding with the introduction of more complex molecular architectures such as multifunctional urethane monomers, star-shaped polymer systems, and hybrid resin networks incorporating stress-relieving mechanisms. These more recent studies also increasingly combined shrinkage stress reduction strategies with additional functionalities, including antibacterial activity, bioactive fillers, and improved hydrolytic stability.

### 3.4. Classification of Studies According to Chemical Strategies for Shrinkage Stress Reduction

Across the reviewed literature, stress-reduction strategies could be grouped according to the mechanism used to control stress development during curing.

Thiol-based systems, including thiol-ene and thiourethane chemistries, formed the largest group [20,21,22,23,24,25,26,27,28,29]. Chain transfer and delayed gelation allow the developing network to relax before stress accumulates. Representative systems included methacrylate-thiol-ene ternary formulations, fluorinated thiol-ene composites, and thiourethane oligomers introduced into the matrix or at the filler interface [20,22,23,24,25,26,27,28,29]. Within the respective study designs, reported reductions ranged from approximately 20% to 78% relative to conventional dimethacrylate composites.

Addition-fragmentation chain transfer (AFCT) mechanisms provide a second stress-relief pathway through reversible bond cleavage and reformation during curing. Allyl sulfide- and trithiocarbonate-containing monomers enabled network rearrangement and were associated with stress reduction in selected formulations [30,31,32,33].

Ring-opening or expanding monomers, including spiro-orthocarbonate derivatives, were used to counteract part of the volumetric contraction through molecular expansion [34,35,36,37]. Their stress-reduction effect was generally formulation-dependent and evaluated relative to conventional methacrylate matrices.

Other approaches retained the dimethacrylate framework but modified reactive group density or polymerization kinetics through alternative diluents and high-molecular-weight structures [38,39,40,41,42,43,44,45,46,47]. Reported examples included catechol-derived reactive diluents, bio-based isosorbide monomers, monofunctional acrylate diluents, PG6EMA, and urethane-based monomers [41,42,43,47,48].

Additional resin systems used alternative polymerization chemistries or hybrid architectures, including ether-based vinyl monomers, nanogel additives, and CuAAC networks [48,49,50,51,52]. Their stress-reduction rationale was delayed gelation, altered kinetics, and/or network rearrangement.

**Figure 3:**
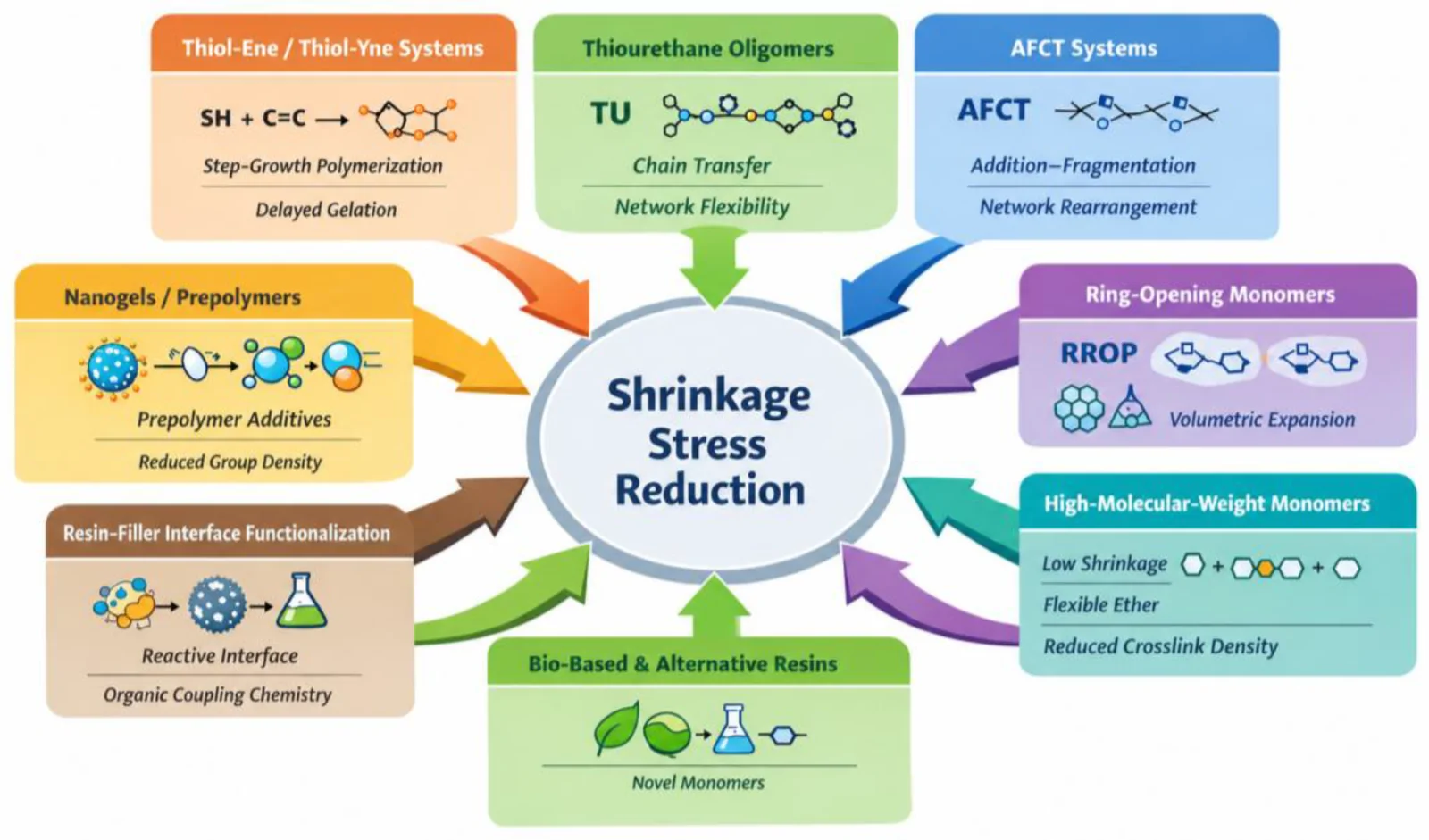
Original schematic overview created by the authors to summarize the main chemical strategies investigated for reducing polymerization shrinkage stress in experimental dental resin-based materials.

Taken together, the literature indicates that several chemistry-based routes can lower polymerization shrinkage stress, but the magnitude of the effect depends on polymerization mechanism, monomer architecture, filler incorporation, and test configuration. Figure 3 summarizes these strategies.

### 3.5. Methods Used to Measure Polymerization Shrinkage Stress

Several techniques were used to measure polymerization shrinkage stress. They differ in specimen configuration, boundary conditions, system compliance, and the measured physical parameter, which helps explain the range of reported values.

The Bioman cantilever beam method was the most common approach. It records the force generated as a contracting specimen deflects a calibrated cantilever, enabling real-time stress monitoring. It was used across thiourethane, AFCT, alternative diluent, and ether-based systems [23,24,25,26,27,28,29,30,31,46,48,49,50], with values typically reported in MPa.

Tensometer systems, including the device developed by the American Dental Association Health Foundation (ADAHF), measure stress generated between two rigid substrates during photopolymerization. By maintaining a controlled configuration factor (C-factor), they support standardized within-method comparisons and were used for thiol-ene, AFCT, nanogel, and CuAAC-based systems [20,21,30,32,51,52].

Universal testing machines adapted for shrinkage-stress measurements were another common option. In these setups, the specimen is bonded between rigid substrates connected to a load cell; the recorded force is then converted to stress according to specimen geometry. This approach was used for ring-opening, alternative diluent, and urethane-based monomer systems [35,38,39,43,47].

Commercial polymerization stress analyzers, including the Proto-Tech Polymerization Stress Tester and the Bisco Shrinkage Stress Analyzer, were also used to evaluate catechol-derived, bio-based, and related modified monomer systems [40,41,42,45].

**Figure 4:**
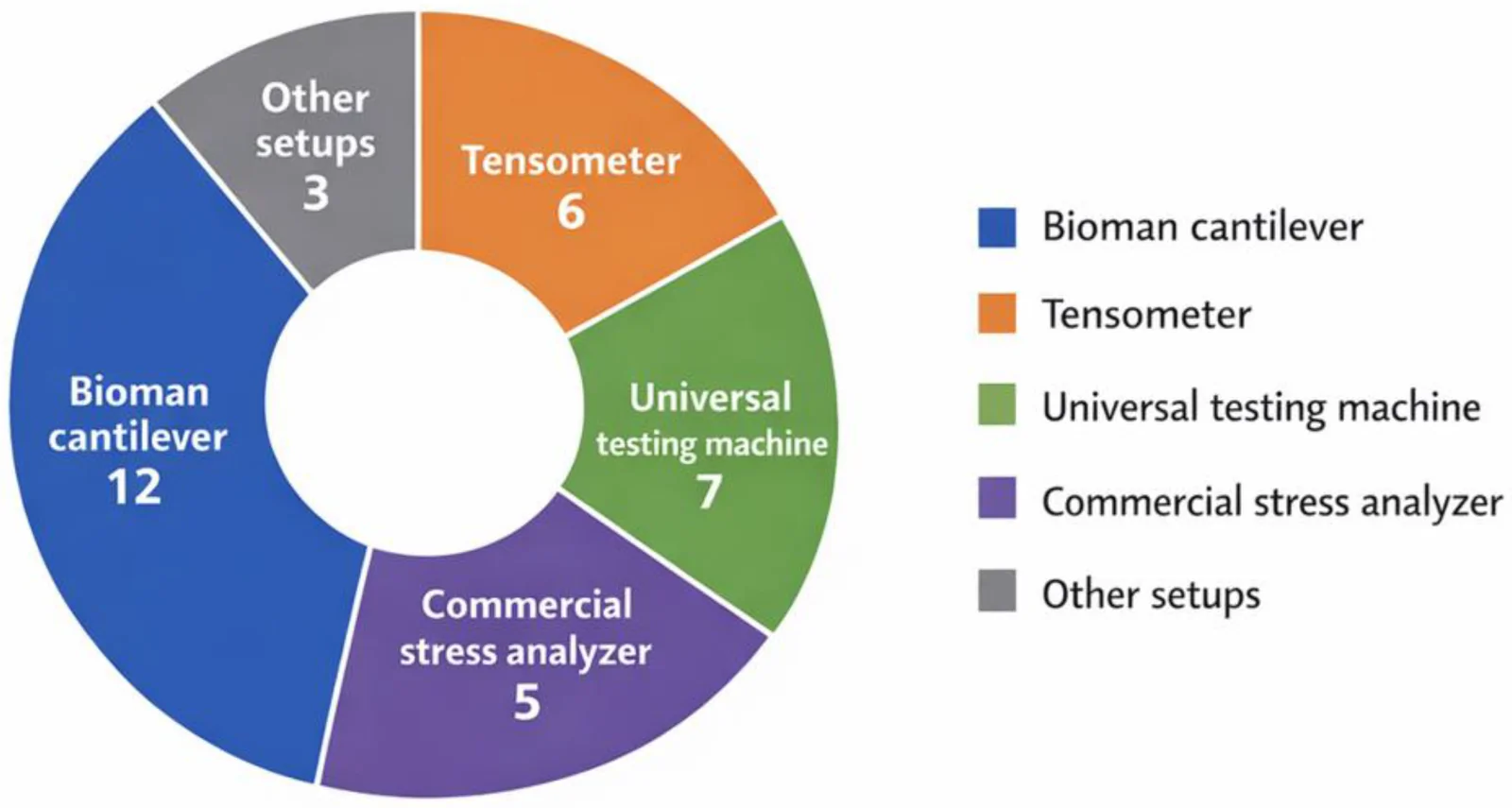
Distribution of measurement methods used to evaluate polymerization shrinkage stress in the included studies.

Because each method imposes different compliance, geometry, curing, and calculation conditions, numerical values should be compared primarily within the same study. Despite this limitation, several experimental resin systems reduced stress relative to their own dimethacrylate controls. Figure 4 summarizes the methods used.

### 3.6. Descriptive Interpretation of Reported Polymerization Shrinkage Stress Outcomes

The included studies reported heterogeneous polymerization shrinkage stress outcomes, reflecting differences in resin formulation, specimen geometry, instrument compliance, C-factor, irradiance, curing protocol, measurement duration, reporting units, and control materials. For this reason, shrinkage stress values were not pooled, converted, or directly compared across studies. Instead, the reported outcomes were interpreted descriptively within the context of each individual experimental design.

Within-study comparisons generally indicated that several experimental chemical strategies were associated with lower polymerization shrinkage stress than their corresponding conventional dimethacrylate controls. Thiol-based and thiourethane-containing systems often showed reduced stress within their respective formulations, mainly through delayed gelation and stress relaxation. AFCT-based systems were associated with stress reduction in selected studies by enabling network rearrangement during polymerization. Ring-opening or expanding monomers showed potential to reduce stress by partially compensating for volumetric contraction, although their performance depended strongly on formulation and conversion behavior. High-molecular-weight monomers, alternative reactive diluents, ether-based systems, and nanogel-modified formulations also showed stress-reduction potential in specific experimental settings.

Because the absolute stress values were generated using different experimental setups, they should be interpreted as study-specific outcomes rather than as directly comparable indicators of material performance. Therefore, the synthesis emphasizes relative trends within individual studies and avoids ranking chemical strategies based on absolute stress values reported by different methodologies.

### 3.7. Methodological Reporting Quality Assessment

The methodological reporting quality assessment showed that the included studies generally provided adequate information regarding resin formulation and shrinkage stress measurement methods. However, reporting was less consistent for some methodological domains, particularly photopolymerization parameters, specimen configuration, measurement time points, and statistical details. Therefore, several studies were classified as having Unclear concern in one or more domains. The overall judgments should be interpreted as indicators of reporting completeness and methodological transparency, rather than as formal risk-of-bias judgments. The detailed assessment is presented in Table 4.

### 3.8. Summary of Included Studies

A concise synthesis of the included studies is presented in Table 4, organized according to the main chemical strategies investigated for reducing polymerization shrinkage stress. Readers interested in the study-level numerical outcomes should consult Supplementary Table S2, which provides the experimental monomers, tested systems, filler-related characteristics, measurement methods, reported shrinkage stress or force values, and main conclusions for each included study. The shrinkage stress values reported in Supplementary Table S2 are presented descriptively in the original units used by each study and should not be interpreted as directly comparable across studies because of differences in testing methods, specimen configuration, curing conditions, reporting units, and measurement time points.

## 4. Discussion

In the studies considered in this scoping review, the investigated strategies aimed either to delay gelation, increase network relaxation after gelation, compensate for volume loss through ring-opening reactions, or reduce reactive group density using monomers with higher molecular weights or prepolymerized additives such as nanogels [53,54,55,56]. Because this review was designed as a scoping review, the findings should be interpreted as a mapping of experimental chemical strategies rather than as a comparative effectiveness assessment. The included studies differed substantially in resin composition, filler loading, control materials, specimen geometry, curing protocols, measurement devices, reporting units, and time points. Therefore, direct quantitative ranking of the investigated monomer systems was not performed. Step-growth and adaptive networks were described as promising approaches within individual experimental designs [22,38,40], while replacement of reactive diluents and nanogel-based formulations were also associated with stress reduction under specific study conditions [47,49,50]. Overall, the synthesis was aligned with the broader mechanistic interpretation of shrinkage stress reported in previous reviews [55], but no definitive hierarchy between strategies was established.

Reported polymerization shrinkage stress values should be interpreted cautiously because they are highly dependent on the experimental setup. Tensometer design, system compliance, specimen dimensions, bonded surface configuration, C-factor, irradiation protocol, measurement duration, and reporting unit can all influence the recorded values. Therefore, MPa and N values from different studies were not treated as equivalent measures for direct comparison; interpretation was restricted to within-study trends and comparisons with the corresponding control materials. Within individual studies, lower shrinkage stress or force values may indicate a reduced tendency for stress transfer to bonded interfaces, which may be consistent with improved marginal integrity and a lower propensity for interfacial debonding or microcrack development. However, these outcomes should be regarded as cautious mechanistic indicators rather than direct predictors of clinical performance.

Mechanistically, the identified strategies are not interchangeable. Thiol-ene, thiol-yne, and thiourethane systems mainly reduce stress by delaying gelation, promoting chain-transfer reactions, and allowing greater network relaxation during polymerization [29]. Their practical effect depends on oligomer concentration, viscosity, filler incorporation, and resin-filler interface compatibility. AFCT-based systems provide stress relief through reversible bond exchange and network rearrangement [30,31,32]. Although this mechanism can produce marked stress reduction in selected systems, translation may be constrained by synthesis complexity, formulation sensitivity, and the need to maintain adequate polymerization kinetics and mechanical performance.

Ring-opening or -expanding monomers, including spiro-orthocarbonate-related systems, aim to compensate for volumetric contraction through molecular expansion [34,35,36,37]. However, their effectiveness depends on the extent of ring-opening reaction, compatibility with methacrylate networks, degree of conversion, and final mechanical properties. High-molecular-weight monomers, alternative reactive diluents, ether-based systems, and nanogel-modified formulations generally reduce stress by lowering reactive group density, modifying polymerization kinetics, or introducing prepolymerized stress-relieving structures [38,39]. These approaches may offer formulation flexibility, but they also require careful balancing of viscosity, handling, conversion, mechanical properties, and long-term stability.

The most robust results in filled composite systems come from thiol-based chain-transfer chemistries. In methacrylate–thiolene formulations, the stress reduction is attributed to slower kinetics and high conversion, as observed in Boulden’s [20], Podgórski’s ester-free systems [21], and Fu’s fluorinated thiolene systems [22]. Thiourethane oligomers (added to the matrix or at the filler–resin interface) have provided substantial reductions, frequently 25–60% and sometimes > 70%, along with increased fracture toughness [24,25,26,27,28]. However, studies indicate a formulation threshold: excessively high fractions may increase viscosity [57].

Mechanically, shrinkage stress is determined not only by volumetric shrinkage but also by the increase in elastic modulus and the degree of constraint of the specimen (C-factor/compliance) [57,58,59]. Classical models distinguish between a pre-gel window (when the material can flow and relax) and a post-gel/post-vitrification window (when relaxation becomes limited), and stress accumulates rapidly as the network enters the vitrified state [60,61]. This framework explains why methods focused solely on light-curing protocol, such as soft-start, have variable and, frequently, more modest effects than chemical interventions, a conclusion consistent with meta-analyses on photoactivation strategies [5]. In our set, step-growth strategies (thioenes, CuAAC) and those involving transfer/fragmentation (thiourethane, AFCT) function by shifting gelation toward higher conversions and through internal dissipation mechanisms. In conclusion, “useful slowing” must be correlated with maintaining conversion and properties, not just with reducing shrinkage stress [62,63].

The most spectacular effects occur when the polymerization mechanism allows for bond rearrangement during curing [64]. In AFCT/RAFT networks, units such as allyl sulfides, β-allyl sulfones, or trithiocarbonates introduce radical bond exchanges that relieve stress; Park et al. [32] demonstrated dramatic reductions in stress in model systems, and Faria-e-Silva et al. [31] applied similar concepts to reinforced composites. The combination of radical ring-opening (vinylcyclopropane) with AFCT (EVS) in Schoerpf [33] shows that mechanistic synergies can broaden the relaxation window. In parallel, spiro-orthocarbonate monomers [34,35,36,37] reduce stress through partial expansion upon ring-opening [38]. However, these solutions are formulation-sensitive: reaction rate and filler compatibility may limit clinical applicability [64].

The heterogeneity of methods explains why absolute stress ranges differ between laboratories. In the included studies, the cantilever method (Bioman) predominates, followed by the strain gauge for stress kinetics and setups on universal testing machines or commercial analyzers [65,66,67,68]. These devices differ in terms of assembly stiffness (compliance), specimen geometry, clamping/adhesion conditions, and the method of converting force to stress (MPa vs. N). For example, within the same monomer families, values obtained on the Bioman tend to be lower than those obtained in very rigid configurations. Thus, quantitative comparisons should preferably be made within the same method and with the same dimethacrylate “control”. Thus, for transparency, authors should report stress-time curves, system compliance, and photopolymerization parameters [69,70,71].

A clear signal from our dataset is the lack of a single “currency”: some studies report MPa, others report force (N), and the reading time (e.g., 5–10 min vs. 60 min) varies. This becomes critical when comparing the values obtained with Proto-Tech [41,42] in the González-López 2020 studies or the BISCO values in Bergeron [45], with the lower values obtained on Bioman in the thiourethane series or in the UDMA/TEG-DVBE systems [48,49].

Furthermore, light curing (irradiance, radiant exposure, spectral match) and the curing method can significantly alter stress kinetics, and Zhang [43] shows that changes in crosslink density can simultaneously alter both shrinkage and modulus. Therefore, for future meta-analyses, the community would benefit from harmonized protocols (geometry, C-factor, simultaneous conversion, reporting in MPa) and the inclusion of a common reference material [72,73,74].

From a clinical perspective, shrinkage stress is associated with marginal gaps, cusp deflection, and postoperative sensitivity, especially in cavities with a high C-factor, according to clinical-mechanical reviews on the consequences of shrinkage stress. However, the included studies suggest that chemical interventions may be more robust than protocol adjustments, as they act on the network formation mechanism. Relevant advances for translation include multifunctional strategies: the UDMA/TEG-DVBE matrix [49,50,75] has been extended with antibacterial monomers [20,76] or remineralizing filler and DMAHDM [49], achieving stress reductions alongside anti-caries functions. Furthermore, high-molecular-weight monomers [46] can reduce stress without major procedural changes. However, prior to clinical application, data on hydrothermal stability, fatigue, adhesion, and performance in actual restorations, not just in standardized tests, are required [77,78,79].

The broad range of reported stress values also reflects formulation factors beyond the resin matrix. Filler loading, filler type, particle size, filler-matrix coupling, viscosity, elastic modulus development, degree of conversion, and curing conditions may all affect stress generation and transfer. Thus, although the review focused on monomer- and matrix-related strategies, differences in composite composition and testing conditions likely contributed to outcome variability.

Several resin systems reduced stress through altered polymerization kinetics, delayed gelation, enhanced relaxation, or lower modulus development rather than through a proportional decrease in volumetric shrinkage. For this reason, shrinkage stress was interpreted separately from volumetric shrinkage, elastic modulus, and degree of conversion.

The clinical relevance of these findings should be interpreted cautiously. Although reduced polymerization shrinkage stress may contribute to improved marginal integrity and lower stress transfer at the tooth–restoration interface, most included studies were performed under simplified in vitro conditions. Therefore, lower shrinkage stress alone does not necessarily indicate superior clinical performance. Future studies should also assess degree of conversion, mechanical properties, adhesion, biocompatibility, aging behavior, and long-term chemical, physical, and mechanical stability.

Commercial translation of these experimental systems also remains challenging. Many low-stress monomer systems require complex synthesis, strict purification procedures, or specialized formulation steps that may limit scalability and increase production costs. In addition, new resin chemistries must be compatible with existing filler technologies, photoinitiator systems, curing lights, clinical handling requirements, and storage conditions. Regulatory approval also requires extensive evidence regarding toxicological safety, leachable components, biocompatibility, batch reproducibility, mechanical durability, and long-term intraoral stability. These factors may explain why several promising experimental systems have not yet progressed to commercial dental restorative materials despite favorable in vitro shrinkage stress results.

As a scoping review, this synthesis aimed to map strategies, not to estimate a uniform effect size (no meta-analysis was performed), and methodological heterogeneity limits numerical comparisons between studies. Furthermore, some articles reported only “significant reduction” without complete values, and units (MPa vs. N) and measurement times varied, which may over- or underestimate the benefits of certain monomers. The selection was restricted to studies with experimental monomers and directly measured stress data, excluding part of the literature on light-curing protocols or commercial products. A further limitation is that the included studies did not consistently classify the tested materials as regular incremental-fill, bulk-fill, paste-like, or flowable formulations. Therefore, subgroup comparisons according to clinical handling category were not performed. This heterogeneity should be considered when interpreting the reported polymerization shrinkage stress values.

Because this was a scoping review, studies were not excluded solely on the basis of high risk of bias. Instead, methodological limitations were considered when interpreting the findings, particularly given the heterogeneity in material formulations, testing methods, and reported shrinkage stress outcomes.

For future studies, it is recommended to standardize reporting (compliance, geometry, C-factor, conversion, and time-dependent modulus), evaluate aging in water, and include dental models (Class I/II) to link stress reduction to clinical parameters such as marginal adaptation and fracture resistance.

## 5. Conclusions

Thiol-based systems (thiol–ene and thiourethane chemistries) represented the most extensively investigated strategy and consistently demonstrated substantial reductions in shrinkage stress, primarily due to delayed gelation and step-growth chain-transfer mechanisms.

Adaptive polymerization mechanisms, particularly addition–fragmentation chain transfer (AFCT) networks, showed the greatest potential for stress reduction by enabling dynamic network rearrangement during polymerization.

Alternative molecular design approaches, including ring-opening monomers, high-molecular-weight dimethacrylates, ether-based monomers, and bio-based diluents, achieved moderate shrinkage stress reduction by modifying polymerization kinetics and reactive group density.

Future studies should evaluate these experimental and modified resin systems under clinically relevant conditions, including aging, fatigue, adhesion, biocompatibility, and long-term chemical, physical, and mechanical stability.

## Figures and Tables

**Table 1:** Detailed electronic search strategy and results across databases included in the scoping review.

Database	Search String/Keywords	Filters Applied	Time Span	Records Identified (n)
**PubMed/MEDLINE**	(“dental resin*” OR “dental composite*” OR “restorative resin” OR “adhesive system”) AND (“polymerization shrinkage” OR “curing contraction” OR “shrinkage stress”) AND (“experimental monomer” OR “novel monomer” OR “alternative co-monomer”)	In vitro studies; English abstract	2010–2025	612
**Scopus**	TITLE-ABS-KEY (“dental resin” OR “dental composite”) AND (“polymerization shrinkage” OR “shrinkage stress”) AND (“experimental formulation” OR “novel monomer”)	Articles and conference papers	2010–2025	488
**Web of Science Core Collection**	(“dental composite*” OR “restorative resin”) AND (“polymerization shrinkage” OR “curing contraction”) AND (“novel monomer” OR “polymer network modification”)	Refined by Dentistry and Materials Science	2010–2025	142
**Embase**	(“dental resin” OR “restorative composite”) AND (“polymerization shrinkage” OR “stress”) AND (“new formulation” OR “alternative co-monomer”)	Experimental studies; English abstract	2010–2025	236
**Google Scholar**	“low-shrinkage dental monomer” OR “experimental monomer dental composite”	First 200 relevant results screened	2010–2025	200
**Total records identified**	–	–	–	1678
**Records after duplicate removal**	–	–	–	1184

**Table 2:** Data extraction template applied in the present review.

Category	Extracted Details
Study identification	First author and year of publication
Study type	In vitro experimental study
Dental material type	Resin-based composite, resin cement, or experimental resin matrix
Experimental monomer(s)/resin system	Newly developed monomer, co-monomer, oligomeric additive, or modified resin system
Chemical strategy	Molecular or resin-matrix-related strategy for polymerization shrinkage stress reduction
Tested system/formulation type	Unfilled resin matrix, filled resin-based composite, resin cement, or resin–filler interface-functionalized system
Filler-related information	Filler type, filler loading, particle size, and surface/interface functionalization, when reported
Shrinkage outcome	Polymerization shrinkage stress
Measurement method	Tensometer, cantilever beam system, universal testing machine, Bioman, or other reported method
Reported values	Shrinkage stress values, reported in MPa, N, or other original units
Main conclusion	Main finding related to polymerization shrinkage stress reduction

**Table 3:** Methodological reporting quality assessment of the included studies.

Study	Resin Formulation Description	Polymerization Protocol	Shrinkage Stress Measurement	Statistical Analysis	Overall
Boulden et al., 2011 [20]	Low	Unclear	Low	Low	Low
Podgórski et al., 2015 [21]	Low	Unclear	Unclear	Low	Unclear
Fu et al., 2019 [22]	Low	Unclear	Low	Low	Low
Bacchi et al., 2015 [23]	Low	Low	Low	Low	Low
Bacchi et al., 2016 [24]	Low	Low	Low	Low	Low
Bacchi et al., 2018 [25]	Low	Low	Low	Low	Low
Fugolin et al., 2018 [26]	Low	Low	Low	Low	Low
Lewis et al., 2019 [27]	Low	Low	Low	Unclear	Low
Fugolin et al., 2021 [28]	Low	Low	Low	Low	Low
Goulard et al., 2022 [29]	Low	Low	Low	Unclear	Low
Park et al., 2012 [30]	Low	Unclear	Low	Low	Low
Faria-e-Silva et al., 2019 [31]	Low	Low	Low	Low	Low
Park et al., 2020 [32]	Low	Low	Unclear	Low	Low
Schoerpf et al., 2016 [33]	Low	Unclear	Unclear	Low	Unclear
Sun et al., 2011 [34]	Low	Unclear	Unclear	Low	Unclear
Fu et al., 2014 [35]	Low	Low	Low	Unclear	Low
Berlanga et al., 2019 [36]	Low	Unclear	Low	Unclear	Unclear
Podgorski et al., 2015 [37]	Low	Unclear	Low	Unclear	Unclear
He et al., 2018 [38]	Low	Low	Low	Low	Low
He et al., 2019 [39]	Low	Low	Low	Low	Low
Herrera-González et al., 2019 [40]	Low	Low	Low	Unclear	Low
González-López et al., 2020 [41]	Low	Low	Low	Low	Low
González-López et al., 2020 [42]	Low	Low	Low	Unclear	Low
Zhang et al., 2020 [43]	Low	Low	Unclear	Unclear	Unclear
He et al., 2020 [44]	Low	Low	Low	Low	Low
Bergeron et al., 2017 [45]	Low	Unclear	Low	Low	Low
Lucena et al., 2024 [46]	Low	Low	Low	Low	Low
Luo et al., 2019 [47]	Low	Low	Low	Low	Low
Wang et al., 2017 [48]	Low	Low	Unclear	Low	Low
Filemban et al., 2022 [49]	Low	Low	Low	Low	Low
Alhussein et al., 2021 [50]	Low	Low	Low	Unclear	Low
Moraes et al., 2011 [51]	Low	Unclear	Unclear	Low	Unclear
Song et al., 2016 [52]	Low	Unclear	Low	Low	Low

Low = adequately reported; Unclear = incompletely reported but interpretable; The overall judgment was based on the combination of individual domains and was used only to support interpretation, not to exclude or weight studies.

**Table 4:** Summary of the main chemical strategies investigated for reducing polymerization shrinkage stress in experimental and modified dental resin-based materials.

Chemical Strategy	Main Mechanism	Tested Systems	General Trend Reported	Key Limitation
Thiol-based systems/thiourethanes	Delayed gelation and stress relaxation	Resin matrices, composites, resin–filler interface-functionalized systems	Frequently associated with reduced shrinkage stress within individual studies	Formulations and testing methods varied
AFCT/addition–fragmentation systems	Network rearrangement during polymerization	Experimental resin systems and composites	Reported reductions in selected formulations	Limited number of comparable studies
Ring-opening systems	Compensation of volumetric contraction	SOC, oxirane, silorane, or related systems	Potential stress reduction depending on formulation	Not always associated with optimal conversion or mechanical behavior
High-molecular-weight or alternative monomers	Reduced reactive group density and altered kinetics	Modified resin matrices or composites	Reduced stress in some experimental systems	Mechanical and handling properties require further validation
Nanogel-modified systems	Prepolymerized stress-relieving additives	Experimental composites or resin matrices	Associated with stress reduction in specific formulations	Long-term stability and clinical relevance remain uncertain

## Data Availability

All data is contained within the article.

## Supplementary Materials

The following supporting information can be downloaded at: https://www.mdpi.com/article/10.3390/dj14080479/s1: Supplementary Table S1. Complete database search strategies. Supplementary Table S2. Detailed data extraction table of the included studies. Values are reported as presented in the original studies and are intended for descriptive mapping only; direct cross-study comparison was not performed.
