# Supplementary material for: Chemical Strategies for Reducing Polymerization Shrinkage Stress in Experimental Dental Resin-Based Materials: A Scoping Review
Source: Dent J (Basel). 2026 Aug 5;14(8):479. doi: 10.3390/dj14080479 (PMC13511717; doi:10.3390/dj14080479)
Supplement: Supplementary file 1 [file dentistry-14-00479-s001.zip › dentistry-4313977-supplementary.pdf]

Supplementary Table S1. Complete database search strategies

PubMed/MEDLINE

**Complete search strategy**	(("Dental Materials"[Mesh] OR "Composite Resins"[Mesh] OR dental composite*[tiab] OR resin composite*[tiab] OR dental resin*[tiab] OR resin-based material*[tiab] OR restorative material*[tiab]) AND (polymerization shrinkage stress[tiab] OR polymerisation shrinkage stress[tiab] OR shrinkage stress[tiab] OR contraction stress[tiab] OR polymerization stress[tiab] OR polymerisation stress[tiab]) AND (experimental monomer*[tiab] OR modified monomer*[tiab] OR resin matrix[tiab] OR thiourethane[tiab] OR thiol-ene[tiab] OR thiolene[tiab] OR thiol-yne[tiab] OR thiolyne[tiab] OR addition-fragmentation[tiab] OR addition fragmentation[tiab] OR AFCT[tiab] OR allyl sulfide[tiab] OR trithiocarbonate[tiab] OR ring-opening[tiab] OR spiro-orthocarbonate[tiab] OR spiroorthocarbonate[tiab] OR silorane[tiab] OR oxirane[tiab] OR vinylcyclopropane[tiab] OR nanogel[tiab] OR low-shrinkage resin[tiab] OR stress-relieving monomer*[tiab]))
**Limits/filters**	No date restriction. Article type filters were not applied during the main search. Non-primary publications were excluded during screening according to eligibility criteria.
**Documentation details**	Search date: [insert date]. Records retrieved: [insert number].

Scopus

**Complete search strategy**	TITLE-ABS-KEY(("dental composite*" OR "resin composite*" OR "dental resin*" OR "resin-based material*" OR "restorative material*") AND ("polymerization shrinkage stress" OR "polymerisation shrinkage stress" OR "shrinkage stress" OR "contraction stress" OR "polymerization stress" OR "polymerisation stress") AND ("experimental monomer*" OR "modified monomer*" OR "resin matrix" OR thiourethane OR "thiol-ene" OR thiolene OR "thiol-yne" OR thiolyne OR "addition-fragmentation" OR "addition fragmentation" OR AFCT OR "allyl sulfide" OR trithiocarbonate OR "ring-opening" OR "spiro-orthocarbonate" OR spiroorthocarbonate OR silorane OR oxirane OR vinylcyclopropane OR nanogel OR "low-shrinkage resin" OR "stress-relieving monomer*"))
**Limits/filters**	No date restriction. Document type filters: articles and reviews were retrieved for sensitivity; reviews and conference abstracts were excluded during screening when they did not meet eligibility criteria.
**Documentation details**	Search date: [insert date]. Records retrieved: [insert number].

Web of Science Core Collection

**Complete search strategy**	TS=(("dental composite*" OR "resin composite*" OR "dental resin*" OR "resin-based material*" OR "restorative material*") AND ("polymerization shrinkage stress" OR "polymerisation shrinkage stress" OR "shrinkage stress" OR "contraction stress" OR "polymerization stress" OR "polymerisation stress") AND ("experimental monomer*" OR "modified monomer*" OR "resin matrix" OR thiourethane OR "thiol-ene" OR thiolene OR "thiol-yne" OR thiolyne OR "addition-fragmentation" OR "addition fragmentation" OR AFCT OR "allyl sulfide" OR trithiocarbonate OR "ring-opening" OR "spiro-orthocarbonate" OR spiroorthocarbonate OR silorane OR oxirane OR vinylcyclopropane OR nanogel OR "low-shrinkage resin" OR "stress-relieving monomer*"))
**Limits/filters**	No date restriction. Search performed in title, abstract, author keywords, and Keywords Plus via Topic Search.
**Documentation details**	Search date: [insert date]. Records retrieved: [insert number].

Google Scholar (supplementary search)

**Complete search strategy**	Primary supplementary query: "dental composite" "polymerization shrinkage stress" "experimental monomer". Additional targeted queries were used to verify retrieval of chemistry-specific approaches: "dental composite" "shrinkage stress" thiourethane; "dental composite" "shrinkage stress" "thiol-ene"; "dental composite" "shrinkage stress" "thiol-yne"; "dental composite" "shrinkage stress" AFCT; "dental composite" "shrinkage stress" "addition-fragmentation"; "dental composite" "shrinkage stress" "ring-opening"; "dental composite" "shrinkage stress" spiroorthocarbonate; "dental composite" "shrinkage stress" nanogel; "dental composite" "shrinkage stress" oxirane.
**Limits/filters**	First 200 results screened, sorted by relevance. Searches were performed without date restriction. Google Scholar was used only as an additional source to identify potentially missed records and citation-linked studies.
**Documentation details**	Search date: [insert date]. Browser/search settings: [insert details if documented]. Records screened: first 200 results. Records added after deduplication: [insert number].

Backward/forward citation checking

**Complete search strategy**	Reference lists of included articles and relevant reviews were manually screened. Forward citation tracking was used for key included studies when available.
**Limits/filters**	Only primary in vitro studies meeting the predefined eligibility criteria were retained.
**Documentation details**	Date performed: [insert date]. Records identified: [insert number]. Records added after deduplication: [insert number].

Abbreviations: AFCT, addition-fragmentation chain transfer; MeSH, Medical Subject Headings; TS, topic search; TITLE-ABS-KEY, title-abstract-keywords; tiab, title/abstract.

Supplementary Table S2. Detailed data extraction table of the included studies. Values are reported as presented in the original studies and are intended for descriptive mapping only; direct cross-study comparison was not performed.

**Author(s)**	Year	Dental Material Type	**Tested system / formulation type**	**Filler-related information reported**	Experimental Monomer(s) / Resin System	Chemical Strategy for Shrinkage Stress Reduction	Shrinkage Outcome Evaluated	Shrinkage Stress Outcome (reported units)	Measurement Method	Main Conclusion Related to Polymerization Shrinkage Stress
**Boulden et al. [20]**	2011	Resin-based dental composite	Filled experimental resin-based composites; methacrylate–thiol–ene ternary systems compared with dimethacrylate controls	Inorganic glass filler used to similar consistency; 90 wt% 0.4 μm glass + 10 wt% Aerosil OX-50. Approx. filler loading: 72.5 wt% in experimental methacrylate–thiol–ene composites; 73.5 wt% in Bis-GMA/TEGDMA control; 76.0 wt% in EBPADMA/TEGDMA control.	EBPADMA / PETMP / TATATO methacrylate-thiol-ene ternary system	Introduction of a thiol–ene step-growth system into the methacrylate network to delay gelation and reduce shrinkage stress.	Polymerization on shrinkage stress (MPa)	~1.5–1.8 MPa (control: ~2.2–2.3 MPa)	Tensometer (American Dental Association Health Foundation)	Methacrylate–thiol–ene systems exhibited lower shrinkage stress and higher degree of conversion compared with conventional dimethacrylate composites.
**Podgórski et al. [21]**	2015a	Resin-based dental composite	Unfilled experimental resin matrices; ester-free thiol–ene resin development	NA; filler not incorporated in this resin-development study.	Ester-free thiol–ene resin systems based on SiTSH thiol oligomers + allyl monomers (TTT, TENE) compared with BisGMA/TEGDMA control	Use of step-growth thiol–ene polymerization and reduction of ester groups, enabling delayed gelation and lower stress development	Polymerization on shrinkage stress	0.48–1.47 MPa (thiol–ene systems) vs 1.16 MPa (BisGMA/TEGDMA control) depending on formulation	Tensometer (American Dental Association Health Foundation)	Thiol–ene networks exhibit lower shrinkage stress at comparable conversion due to delayed gel point and step-growth polymerization mechanism, although stress values depend strongly on formulation.
**Fu et al. [22]**	2020	Resin-based dental composite	Filled experimental resin-based composites; thiol–ene–methacrylate ternary composites	65 wt% silanated barium borosilicate glass filler; particle size NR.	Bis-GMA/TEGDMA methacrylate resin modified with a thiol-ene system composed of fluorinated allyl ether (FUAE) and pentaerythritol tetra(3-mercaptopropionate) (PETMA). The thiol-ene resin was incorporated into the methacrylate matrix at 10–30 wt% to form thiol-ene-	Chain-transfer reactions and delayed gelation through thiol-ene chemistry	Polymerization on shrinkage stress (MPa)	RC-0 (control) ≈ 2.9 MPa<br>RC-10 ≈ 2.4 MPa<br>RC-20 ≈ 2.1 MPa<br>RC-30 ≈ 1.5 MPa<br>Shrinkage stress decreased	Universal testing machine	The incorporation of a thiol-ene-methacrylate ternary resin system containing FUAE significantly reduced polymerization shrinkage stress compared with conventional methacrylate composites, with higher

					methacrylate ternary composites (RC-10, RC-20, RC-30).			progressively with increasing thiol-ene content.		thiol-ene concentrations producing the lowest stress values
**Bacchi et al. [23]**	2015	Resin-based dental composite	Experimental light-cured resin cements modified with thiourethane oligomers	Experimental cements: 25 wt% filler, composed of 15% OX-50 silica, 0.04 μm, and 85% barium glass, 0.7 μm.	Methacrylate resin modified with thiol-ene monomers (PETMP and TMP derivatives)	Thiol-ene chain transfer delaying gelation and allowing stress relaxation	Polymerization shrinkage stress (MPa)	**3.15–6.86 MPa vs 13.5 MPa control (~49–77% reduction)**	Cantilever system (Bioman)	Incorporation of thio-urethane oligomers significantly reduced polymerization stress while improving fracture toughness and maintaining degree of conversion.
**Bacchi et al. [24]**	2016	Resin-based dental composite	Experimental composite cements modified with thiourethane oligomers	60 wt% barium glass filler, 0.7 μm.	Methacrylate resin modified with thiourethane oligomers (aromatic and aliphatic TU)	Stress relaxation via thiol chain-transfer reactions during polymerization	Polymerization shrinkage stress (MPa)	**11.6–15.3 MPa vs 20.7 MPa control (~26–44% reduction)**	Cantilever system (Bioman)	Thio-urethane modified composites showed reduced polymerization stress without compromising conversion or mechanical properties.
**Bacchi et al. [25]**	2018	Resin-based dental composite	Filled experimental restorative composites modified with thiourethane oligomers in the resin matrix	70 wt% filler, composed of 7% OX-50 silica, 0.04 μm, and 93% silica/barium glass filler, 0.7 μm.	Thio-urethane oligomer-modified methacrylate resin system	Addition of high-molecular-weight thiol-urethane oligomers to reduce polymerization rate and stress development	Polymerization shrinkage stress (MPa)	≈40% reduction in shrinkage stress vs control	Cantilever system (Bioman)	Thio-urethane incorporation consistently reduced polymerization stress while maintaining mechanical properties of the composite system.
**Fugolin et al. [26]**	2021	Resin-based dental composite	Filled experimental composites with thiourethane-treated filler interface	50 wt% barium silicate glass filler, average size 1.0 μm; filler surface functionalized with thiourethane silane.	Methacrylate resin modified with thiourethane oligomers (PETMP and TMP derivatives)	Thiol-mediated chain transfer enabling delayed gelation and stress relaxation	Polymerization shrinkage stress (MPa)	**6.7–11.9 MPa vs 15.5 MPa control (~23–57% reduction)**	Cantilever system (Bioman)	Thiourethane-functionalized fillers significantly reduced polymerization stress while increasing fracture toughness of composites.
**Lewis**	201	Resin-	Filled experimental	70 wt% total inorganic filler;	Bis-GMA / UDMA /	Addition of thiol-	Polymerizati	0.6–1.7 MPa vs	Cantilever	Thiourethane oligomers

**et al. [27]**	9	based dental composite	composites with varied proportions of TU-silanized and methacrylate-silanized fillers	5 wt% Aerosil OX-50 fumed silica and 95 wt% 0.7 μm silica/barium glass filler. TU-silanized filler replaced methacrylate-silanized filler in different proportions.	TEGDMA resin containing thiourethane oligomers synthesized from PETMP and di-isocyanates, incorporated as matrix modifiers	containing oligomers promoting chain-transfer reactions during polymerization	on shrinkage stress (MPa)	2.9 ± 0.3 MPa control (≈40–78% reduction)	system (Bioman)	delayed gelation and reduced polymerization stress without compromising conversion.
**Fugolin et al. [28]**	2018	Resin-based dental composite	Filled experimental composites with systematically varied filler size, loading, and surface treatment	Barium-alumino silicate fillers of 1, 3, and 10 μm; filler loading varied at 50, 60, and 70 wt%; fillers treated with TU-silane, MA-silane, or left untreated.	Methacrylate resin modified with thiourethane oligomers (PETMP and TMP derivatives)	Thiol-mediated chain transfer enabling delayed gelation and stress relaxation	Polymerization shrinkage stress (MPa)	41–54% reduction in polymerization stress vs methacrylate-silane fillers	Cantilever system (Bioman)	Addition of thiourethane oligomers reduced polymerization stress and improved fracture toughness of resin composites.
**Goulard et al. [29]**	2022	Resin-based dental composite	Filled experimental composites with thiourethane-silane filler functionalization	50 wt% Ba-Al-SiO_2 glass filler; average particle size 1.5 μm; fillers silanized with 1, 2, or 4 wt% thiourethane silane.	Composite with fillers functionalized using thiourethane oligomers (BDI or HDDI silanes)	Thiol-mediated chain transfer at the filler interface to reduce stress development	Polymerization shrinkage stress (MPa)	**4.6–8.6 MPa vs 9.5 MPa control (~9–52% reduction)**	Cantilever system (Bioman)	Thiourethane oligomers significantly reduced polymerization stress without compromising mechanical properties.
**Park et al. [30]**	2012	Resin-based dental composite	Filled experimental resin-based composites; norbornene–methacrylate AFCT systems	75 wt% surface-treated microparticle silica filler; size range 0.01–3.6 μm, average size 0.6 μm.	MDNS / PAS norbornene-methacrylate resin system	Incorporation of allyl sulfide groups to enable an AFCT mechanism, allowing polymer network rearrangement and stress relaxation.	Polymerization shrinkage stress (MPa)	~0.13 MPa (control: ~2.9 MPa)	Tensometer (American Dental Association Health Foundation)	Norbornene–methacrylate networks incorporating an AFCT mechanism exhibited a dramatic reduction in shrinkage stress (~96%) compared with conventional methacrylate systems.
**Faria-e-Silva et al. [31]**	2019	Resin-based dental composite	Filled experimental composites with thiourethane-modified silane/filler interface	70 wt% barium glass filler; average particle size 1 μm; fillers functionalized with 5 wt% TU-silane; MA-silane, SH-silane, and no-silane groups used as controls.	Bis-GMA / UDMA / TEGDMA resin modified with addition–fragmentation chain transfer (AFCT) monomer containing β-allyl sulfone functional group	Incorporation of AFCT monomer enabling bond rearrangement and network relaxation during polymerization	Polymerization shrinkage stress (MPa)	**6.3–9.4 MPa vs 12.2 MPa control (~23–48% reduction)**	Cantilever system (Bioman)	AFCT-modified systems significantly reduced polymerization stress due to stress-relieving bond exchange during polymerization.

**Park et al. [32]**	2020	Resin-based dental composite	Filled experimental resin-based composites; trithiocarbonate dimethacrylate / Bis-GMA systems	75 wt% barium glass filler, 0.4 μm, not surface-treated.	BisGMA–TTCDMA (70/30 wt%) resin composite containing trithiocarbonate dimethacrylate (TTCDMA) compared with conventional BisGMA–TEGDMA composite; composites included 75 wt% silica fillers and BAPO photoinitiator	Incorporation of trithiocarbonate RAFT-capable dimethacrylate (TTCDMA) enabling addition–fragmentation chain transfer (AFCT) reactions that allow network rearrangement and stress relaxation during polymerization	Polymerization shrinkage stress (MPa)	0.6 ± 0.02 MPa (BisGMA–TTCDMA) vs 1.7 ± 0.04 MPa (BisGMA–TEGDMA control) (~65% reduction)	Tensometer	Experimental monomers showed lower shrinkage stress compared with conventional methacrylate matrices while maintaining conversion.
**Schoerpf et al. [33]**	2016	Resin-based dental composite	Filled experimental composite formulations based on VCP/EVS systems	13 g silanized Ba-Al glass filler and 7 g monomer mixture, corresponding to approx. 65 wt% filler; filler particle size 1 μm.	Vinylcyclopropane-based monomers (ADVCP + TEGDVCP) combined with ester-activated vinyl sulfonate (EVS) AFCT reagent; reference: UDMA/TEGDMA methacrylate system	Combination of radical ring-opening polymerization (RROP) of vinylcyclopropanes with addition–fragmentation chain transfer (AFCT) via EVS to delay gelation and regulate network formation	Polymerization shrinkage stress (MPa)	MA reference: ~27.3 N; VCP reference: ~16.3 N; VCP/EVS systems: ~7.95–18.4 N depending on EVS content → substantial reduction vs methacrylate system	Universal testing machine stress analyzer	Combining VCP monomers with AFCT reagent EVS significantly reduces polymerization shrinkage stress, mainly by delaying gelation and enabling higher double-bond conversion, producing more homogeneous polymer networks.
**Sun et al. [34]**	2011	Resin-based dental composite	Filled experimental methacrylate/SOC/epoxy resin composites	74 wt% inorganic filler; filler supplied by Esstech; filler type and particle size NR.	Bis-GMA / epoxy / SOC resin system	Incorporation of a spiro-orthocarbonate expanding monomer into the methacrylate matrix to achieve	Polymerization shrinkage stress (MPa)	~1.86 MPa (control: ~3.56 MPa)	Universal testing machine	Incorporation of the SOC monomer reduced both volumetric shrinkage and contraction stress compared with conventional

						polymerization-induced expansion.				methacrylate composites.
**Fu et al. [35]**	2014	Resin-based dental composite	Filled experimental BisS-GMA/BMSOC/TEGDMA composites	70 wt% inorganic filler; SiO_2 filler larger than 230 mesh; filler modified with KH-570 coupling agent.	BisS-GMA / BMSOC / TEGDMA resin system	Introduction of a spiro-orthocarbonate expanding monomer undergoing ring-opening polymerization to compensate for polymerization shrinkage.	Polymerization shrinkage stress (MPa)	Values not reported	Universal testing machine	The BMSOC monomer, which expands via a ring-opening mechanism, significantly reduced both shrinkage and contraction stress compared with conventional methacrylate composites.
**Berlanga Duarte et al. [36]**	2019	Resin-based dental composite	Filled experimental SOC-UDMA and SOC-IP-UDMA dental composites	SiO_2 filler used as micro/nano mixture: 44.8 wt% micro-SiO_2 and 19.2 wt% nano-SiO_2; total SiO_2 filler 64.0 wt%. Silicon dioxide nanopowder reported as 10–20 nm.	SOC-UDMA and SOC-IP-UDMA spiroorthocarbonate methacrylic monomers incorporated into dental composites and compared with bis-GMA matrix	Expanding monomers (spiroorthocarbonates) undergoing double ring-opening polymerization, producing volumetric expansion that counteracts polymerization shrinkage	Polymerization shrinkage stress	~50% reduction in shrinkage stress compared with bis-GMA control (10 N for SOC-UDMA and 7 N for SOC-IP-UDMA vs 19 N control)	Universal testing machine stress analyzer	SOC-based monomers significantly decreased polymerization shrinkage stress due to ring-opening expansion and higher molecular weight, demonstrating potential as alternative low-shrinkage resin matrices.
Podgórski et al. [37]	2015b	Resin-based dental composite	Filled experimental dental composites; composite-development study	Functionalized Schott glass microparticles; filler loading 50 wt% for TEOC formulations and 65 wt% for TEC formulations. Fillers were allyl-, thiol-, or methacrylate-functionalized depending on the resin	Ester-free thiol–ene composite systems based on SiTSH, TTT, TTTSH/DVS, and TENE; Bis-GMA/TEGDMA 70/30 used as dimethacrylate control	Ester-free step-growth thiol–ene polymerization designed to delay gelation/stress development and improve hydrolytic	Polymerization shrinkage stress	Final shrinkage stress ranged from 0.52–0.63 MPa in 50 wt% filled thiol–ene composites and from 0.45–0.76 MPa in 65 wt%	ADAHF-PRC tensometer; shrinkage stress measured as tensile force generated during polymerization	Ester-free thiol–ene composites reduced polymerization shrinkage stress compared with the Bis-GMA/TEGDMA control, with TEOC 02 and TEC 04 showing the lowest

				system/control. Mean filler particle size reported as 0.4 µm.		stability; thiol-, allyl-, or methacrylate-functionalized glass fillers used according to the resin system		filled thiol–ene composites, compared with 0.81–0.83 MPa for Bis-GMA/TEGDMA controls.	divided by sample cross-sectional area	values.
**He et al. [38]**	2018	Resin-based dental composite	Filled experimental composites with SR833s/IBOA alternative diluent monomers	71.4 wt% silanized $BaAlSiO_2$ filler; particle size 0.7 µm; resin matrix/filler ratio 2/5 wt/wt.	Bis-GMA / SR833s / IBOA resin system	Replacement of the TEGDMA diluent with low-shrinkage acrylate co-monomers to reduce polymerization contraction.	Polymerization shrinkage stress (MPa)	**2.83–3.83 MPa vs 4.75 MPa control (~19–40% reduction)**	Universal testing machine	Replacement of TEGDMA with SR833s and IBOA reduced both shrinkage and shrinkage stress without compromising mechanical properties.
**He et al. [39]**	2019	Resin-based dental composite	Filled experimental composites with Phene-modified Bis-GMA/TEGDMA resin matrix	71 wt% particulate filler; silanized $BaAlSiO_2$ filler particles, 0.7 µm.	Bis-GMA / TEGDMA resin matrix modified with Phene monomer (N-methyl-bis(ethyl-carbamate-isopropyl-methylstyryl)amine)incorporated at 10–40 wt%	Introduction of a bulky aromatic monomer to reduce reactive group density and polymer network mobility.	Polymerization shrinkage stress (MPa)	≈2.8–3.8 MPa vs ≈4.75 MPa control (≈20–40% reduction)	Universal testing machine	Incorporation of the Phene monomer into the Bis-GMA/TEGDMA matrix reduced both volumetric shrinkage and polymerization stress compared with conventional methacrylate systems.
**Herrera-González et al. [40]**	2019	Resin-based dental composite	Filled experimental composites with isosorbide-derived monomers as Bis-GMA diluents	65 wt% silanized inorganic filler; reported as 55 wt% microfiller + 10 wt% nanofiller; specific filler chemistry/particle size NR.	Bis-GMA / IBAC and Bis-GMA / IBM systems, where IBAC = isosorbide bis(allyl carbonate) and IBM = isosorbide bis(methacrylate).	Use of bio-based monomers derived from isosorbide to replace TEGDMA.	Polymerization shrinkage stress (MPa)	BisGMA/TEGDMA – 1,03 Mpa BisGMA/IBAC – 0,71 Mpa BisGMA/IBM – 0,63 MPa	Proto-Tech's Polymerization Stress Tester	Bio-based monomers derived from isosorbide reduced volumetric shrinkage compared with the conventional Bis-GMA/TEGDMA system.
**González-López et al.**	2020	Resin-based dental composit	Filled experimental composites with catechol-derived monomers replacing TEGDMA	65 wt% previously silanized SiO_2 particles; micrometric size.	Bis-GMA/CT-AL and Bis-GMA/CT-ACR systems, based on catechol-derived monomers used as reactive	Înlocuirea TEGDMA cu monomeri catechol-derived	Polymerization shrinkage stress (MPa)	13.2–14.4 MPa vs 26.8 MPa control (≈46–51% reduction)	Proto-Tech's Polymerization Stress Tester	Catechol-derived monomers reduced both volumetric shrinkage and shrinkage stress

**[41]**		e			diluents.	pentru a reduce polymerization stress				compared with conventional methacrylate-based composites.
**González-López et al. [42]**	2020	Resin-based dental composite	Filled experimental composites with BZ-AL allyl carbonate monomer as Bis-GMA diluent	55 wt% previously silanized micron-sized silicon oxide filler.	BZ-AL (allyl(2-(2-(((allyloxy)carbonyl)oxy)benzoyl)-5-methoxyphenyl) carbonate) used as diluent replacing TEGDMA in Bis-GMA matrix	Use of diallyl carbonate monomer with slower polymerization kinetics to reduce shrinkage stress	Polymerization shrinkage stress (MPa)	7.3–10.7 N vs 13.5 N control (~20–46% reduction)	Proto-Tech's Polymerization Stress Tester	Allyl-carbonate monomer BZ-AL reduced polymerization stress by about half while maintaining similar volumetric shrinkage and mechanical properties.
**Zhang et al. [43]**	2020	Resin-based dental composite	Filled experimental composite with BOCNA monovinyl acrylate diluent; compared with commercial composites	Experimental BB/BT composites: 75 wt% barium borosilicate glass, 0.7–2.0 µm. Commercial controls: Tetric N-Ceram 80–81 wt%, Neofil 75 wt%, Gradia Direct 77 wt%.	Bis-GMA matrix where TEGDMA diluent replaced with monovinyl acrylate BOCNA (2-(phenyl amino formaldehyde-based oxygen ethyl methacrylate))	Use of monofunctional acrylate diluent to reduce crosslink density and volumetric shrinkage	Polymerization shrinkage stress (MPa)	2.59–3.02 MPa vs 3.88 MPa control (~22–33% reduction)	Universal testing machine	Replacement of TEGDMA with BOCNA reduced both volumetric shrinkage and shrinkage stress while maintaining mechanical properties.
**He et al. [44]**	2020	Resin-based dental composite	Filled experimental composites with HE-Phene or TE-Phene comonomers	71.4 wt% silanized $BaAlSiO_2$ filler; particle size 0.7 µm; resin matrix/filler ratio 2/5 wt/wt.	Bis-GMA/TEGDMA with HE-Phene or TE-Phene	High-molecular-weight γ-methylstyryl comonomers (Phene-type) to reduce double-bond concentration and polymerization rate.	Polymerization shrinkage stress (MPa)	**0.4–3.5 MPa vs 4.8 MPa control (~27–92% reduction)**	Universal testing machine	Experimental UDMA/TEG-DVBE composites generated significantly lower polymerization stress compared with Bis-GMA/TEGDMA systems while maintaining mechanical performance.
**Bergeron et al. [45]**	2017	Resin-based dental composite	Filled experimental composites with PFOEA fluorinated acrylate and EP5001 oxirane	70 wt% acrylate-silanated barium glass filler; particle size 0.7 µm.	Fluorinated acrylate 2-(perfluorooctyl)ethyl acrylate (PFOEA) and difunctional oxirane EPALLOY™ 5001 (EP5001) incorporated into a Bis-GMA/TEGDMA (70:30)	Incorporation of a fluorinated acrylate and oxirane monomer into a methacrylate resin	Polymerization shrinkage stress (MPa)	Control: 2.17 MPa; experimental composites 1.00–1.54 MPa	Bisco Polymerization Stress Tester	The incorporation of the fluorinated acrylate PFOEA and the oxirane EP5001 significantly reduced polymerization

					resin matrix.	system to increase hydrophobicity and reduce polymerization shrinkage and shrinkage stress.				shrinkage stress compared with the conventional Bis-GMA/TEGDMA control. Experimental composites showed shrinkage stress values of approximately 1.00–1.54 MPa, compared with 2.17 MPa for the control, indicating that this resin modification strategy effectively lowers polymerization-induced stress in dental composites.
**Lucena et al. [46]**	2024	Resin-based dental composite	Experimental adhesive/resin matrices and filled composites based on PG6EMA/TEGDMA	Composite formulations: 70 wt% filler, composed of 5% OX-50, 50 nm, and 95% barium glass, 0.7 μm. Resin-only formulations also tested for kinetics/rheology.	Novel dimethacrylate monomer PG6EMA synthesized from bisphenol-A diglycidyl ether and 4-piperidineethanol, copolymerized with TEGDMA	Replacement of BisGMA with a high molecular weight dimethacrylate monomer (PG6EMA) designed to reduce molar shrinkage and polymerization stress	Polymerization shrinkage stress (MPa)	6.2 ± 0.1 MPa (PG6EMA/TEGDMA) vs 7.5 ± 0.7 MPa (BisGMA/TEGDMA) → ≈18.4% reduction in shrinkage stress	Cantilever system (Bioman)	The PG6EMA-based composite significantly reduced polymerization shrinkage stress compared with the BisGMA/TEGDMA control while maintaining similar stiffness, indicating that the high molecular weight monomer effectively decreases shrinkage stress.
**Luo et al. [47]**	2019	Resin-based dental composite	Filled Bis-GMA-free experimental composites based on synthesized urethane dimethacrylate monomer TMA	71.4 wt% silanated $BaAlSiO_2$ filler; particle size 0.7 μm; resin matrix/filler ratio 2/5 wt/wt.	Synthesized urethane dimethacrylate monomer TMA (derived from isophorone diisocyanate + tricyclo[5.2.1.0(2,6)]decanedimethanol +	Use of high molecular weight urethane dimethacrylate to reduce double bond	Volumetric shrinkage (%) and shrinkage stress (MPa)	UDMA control: 3.6 ± 0.2 MPa • Bis-GMA control: 3.3 ± 0.1 MPa • EC-1: 3.6 ± 0.1 MPa	Universal testing machine shrinkage stress setup	TMA-containing composites showed lower volumetric shrinkage and reduced polymerization stress compared with Bis-

					HEMA) incorporated into UDMA matrix replacing Bis-GMA	concentration and shrinkage		• EC-2: 3.4 ± 0.3 MPa • EC-3: 3.2 ± 0.1 MPa • EC-4: 2.9 ± 0.1 MPa • EC-5: 2.6 ± 0.1 MPa (lowest)		GMA and UDMA controls.
**Wang et al. [48]**	2017	Resin-based dental composite	Filled experimental composites based on UDMA/TEG-DVBE hydrolytically stable resin systems	75 wt% total filler: 67.5 wt% silanized $BaBFAlSiO_4$ milled glass + 7.5 wt% Aerosil OX-50 nanoparticles.	UDMA copolymerized with hydrolytically stable ether monomer TEG-DVBE (UDMA:TEG-DVBE ratios 3:1 and 1:1)	Replacement of ester-containing diluent with ether-based vinyl monomer to slow polymerization and reduce stress accumulation	Polymerization shrinkage stress (MPa)	2.20 MPa (control); 1.72 MPa (U3V1); 1.04 MPa (U1V1) — up to 53% reduction in shrinkage stress.	Cantilever system (Bioman)	Dental composites based on UDMA/TEG-DVBE hydrolytically stable ether-based monomers produced significantly lower polymerization stress than conventional Bis-GMA/TEGDMA composites, likely due to altered polymerization kinetics and delayed gelation, enabling stress relaxation during curing.
**Filemban et al. [49]**	2022	Resin-based dental composite	Bioactive low-shrinkage-stress resin composites containing DMAHDM and NACP	65 wt% total filler: 20 wt% NACP nanoparticles, mean size 116 nm, + 45 wt% silanized barium boroaluminosilicate glass, median size 1.4 μm. Commercial Heliomolar control contained 66.7 wt% silica/ytterbium trifluoride nanofillers.	UDMA + triethylene glycol divinylbenzyl ether (TEG-DVBE) resin with antibacterial monomer DMAHDM and remineralizing filler NACP	Ether-based monomer system delaying gel point and allowing stress relaxation	Polymerization shrinkage stress (MPa)	1.05 ± 0.12 MPa (UV + 5% DMAHDM + NACP) vs 1.69 ± 0.08 MPa (traditional BT resin control)	Cantilever system (Bioman)	The experimental UV resin system significantly reduced polymerization shrinkage stress (~37%) compared with a conventional Bis-GMA/TEGDMA resin while maintaining high conversion (~70%)
**Alhussein et al. [50]**	2023	Resin-based dental composit	Low-shrinkage-stress antibacterial resin composites containing DMADDM	70 wt% silanized barium boroaluminosilicate glass particles; particle size 1.2 μm.	UDMA + TEG-DVBE low-shrinkage resin (UV) with DMADDM (0–5%) and 70% glass fillers	Use of ether-based monomer (TEG-DVBE) combined with UDMA to	Polymerization shrinkage stress (MPa)	1.12 ± 0.06 MPa (UV + 5% DMADDM) vs 2.45 ± 0.14 MPa	Cantilever system (Bioman)	The UDMA/TEG-DVBE low-shrinkage resin significantly reduced polymerization

		e				delay gel point and allow stress relaxation; incorporation of antibacterial DMADDM (QAM monomer)		(BisGMA-TEGDMA control)		shrinkage stress (~50%) compared with conventional BisGMA-TEGDMA composites while maintaining adequate degree of conversion (~62–67%).
**Moraes et al. [51]**	2011	Resin-based dental composite	Nanogel-modified monomer systems and filled composite formulations	Both unfilled TEGDMA/nanogel resin systems and filled composites were tested. Filled composites used fixed 70 wt% barium glass filler; maximum filler loading capacity ranged from 81.7 wt% in control to 75.8 wt% at 40 wt% nanogel.	TEGDMA resin modified with reactive nanogel particles (IBMA-UDMA based nanogels) added at 20–40 wt%; composites also contained barium glass filler	Incorporation of prepolymerized nanogel particles to reduce reactive group concentration and allow network rearrangement during curing	Polymerizati on shrinkage stress	≈26% reduction with 20 wt% nanogel and ≈45% reduction with 40 wt% nanogel compared with control	Tensometer with simultaneous real-time FTIR/NIR monitoring of conversion	Nanogel incorporation significantly reduces polymerization shrinkage stress without affecting conversion or modulus, providing an effective strategy to control stress development in dental composites
**Song et al. [52]**	2016	Resin-based dental composite	Unfilled experimental resin matrices; photo-CuAAC azide–alkyne resin systems compared with Bis-GMA-based resin	NA; filler not incorporated in the polymerization stress experiments reported in the uploaded article.	Photo-CuAAC resin system based on diazide monomers (BZ-AZ, BPA-AZ) + trialkyne (AK) compared with BisGMA/TEGDMA control	Step-growth “click” polymerization (photo-initiated CuAAC – azide-alkyne cycloaddition) leading to delayed gelation and stress relaxation	Polymerizati on shrinkage stress (MPa)	0.27–0.43 MPa (CuAAC systems) vs 1.3 MPa (BisGMA/TEGDMA control)	Tensometer (American Dental Association Health Foundation)	CuAAC step-growth polymerization produced ~3–4× lower shrinkage stress than dimethacrylate resins due to delayed gelation and slower stress development despite similar volumetric shrinkage.

NA = not applicable; NR = not reported

References

20. Boulden, J.E.; Cramer, N.B.; Schreck, K.M.; Couch, C.L.; Bracho-Troconis, C.; Stansbury, J.W.; Bowman, C.N. Thiol–ene–methacrylate ternary networks for dental restorative materials. *Dent. Mater.* **2011**, *27*, 267–272.
21. Podgórski, M.; Becka, E.; Bowman, C.N. Ester-free thiol–ene dental resins with reduced polymerization stress. *Dent. Mater.* **2015**, *31*, 125–133.

22. Fu, W.; Wang, L.; He, J. Evaluation of mechanical properties and shrinkage stress of thiol-ene-methacrylate dental composites with synthesized fluorinated allyl ether. *J. Mech. Behav. Biomed. Mater.* **2019**, *95*, 53–59. https://doi.org/10.1016/j.jmbbm.2019.03.027.
23. Bacchi, A.; Consani, R.L.; Martim, G.C.; Pfeifer, C.S. Thio-urethane oligomers improve the properties of light-cured resin cements. *Dent. Mater.* **2015**, *31*, 565–574.
24. Bacchi, A.; Consani, S.; Pfeifer, C.S. Influence of thiourethane oligomers on polymerization stress and mechanical properties of dental composites. *Dent. Mater.* **2016**, *32*, 978–986.
25. Bacchi, A.; Yih, J.A.; Platta, J.; Knight, J.; Pfeifer, C.S. Shrinkage / stress reduction and mechanical properties improvement in restorative composites formulated with thio-urethane oligomers. *J. Mech. Behav. Biomed. Mater.* **2018**, *78*, 235–240. https://doi.org/10.1016/j.jmbbm.2017.11.011.
26. Fugolin, A.P.; Sundfeld, D.; Ferracane, J.L.; Pfeifer, C.S. Toughening of Dental Composites with Thiourethane-Modified Filler Interfaces. *Sci. Rep.* **2019**, *9*, 2286. https://doi.org/10.1038/s41598-019-39003-w.
27. Lewis, S.; App, F.; Lam, S.; Scanlon, C.; Ferracane, J.; Pfeifer, C. Effects of systematically varied thiourethane-functionalized filler concentration on polymerization behavior and relevant clinical properties of dental composites. *Mater. Des.* **2021**, *197*, 109249. https://doi.org/10.1016/j.matdes.2020.109249.
28. Fugolin, A.P.P.; Costa, A.R.; Correr-Sobrinho, L.; Chaw, R.C.; Lewis, S.; Ferracane, J.L.; Pfeifer, C.S. Toughening and polymerization stress control in composites using thiourethane-treated fillers. *Sci. Rep.* **2021**, *11*, 7638. https://doi.org/10.1038/s41598-021-87151-9.
29. Goulart, M.; Fugolin, A.; Lewis, S.; Rodrigues, J.; Erhardt, M.; Pfeifer, C. Thiourethane filler functionalization for dental resin composites: Concentration-dependent effects on toughening, stress reduction and depth of cure. *Mater. Sci. Eng. C* **2021**, *118*, 111535–111535. https://doi.org/10.1016/j.msec.2020.111535.
30. Park, H.Y.; Kloxin, C.J.; Scott, T.F.; Bowman, C.N. Stress relaxation via addition–fragmentation chain transfer in dental restorative materials. *Macromolecules* **2012**, *45*, 5647–5653.
31. Faria-e-Silva, A.L.; Dos Santos, A.; Tang, A.; Girotto, E.M.; Pfeifer, C.S. Effect of thiourethane filler surface functionalization on stress, conversion and mechanical properties of restorative dental composites. *Dent. Mater.* **2018**, *34*, 1351–1358.
32. Park, H.Y.; Kloxin, C.J.; Abuelyaman, A.S.; Oxman, J.D.; Bowman, C.N. Novel dental restorative materials having low polymerization shrinkage stress via stress relaxation by addition-fragmentation chain transfer. *Dent. Mater.* **2012**, *28*, 1113–1119. https://doi.org/10.1016/j.dental.2012.06.012.
33. Schoerpf, S.; Catel, Y.; Moszner, N.; Gorsche, C.; Liska, R. Enhanced reduction of polymerization-induced shrinkage stress *via* combination of radical ring opening and addition fragmentation chain transfer. *Polym. Chem.* **2019**, *10*, 1357–1366. https://doi.org/10.1039/c8py01540f.
34. Sun, X.; Li, Y.; Xiong, J.; Hu, X.; Chen, J. Shrinkage properties of a modified dental resin composites containing a novel spiro-orthocarbonate expanding monomer. *Mater. Lett.* **2011**, *65*, 3586–3589. https://doi.org/10.1016/j.matlet.2011.07.097.
35. Fu, J.; Liu, W.; Hao, Z.; Wu, X.; Yin, J.; Panjiyar, A.; Liu, X.; Shen, J.; Wang, H. Characterization of a low shrinkage dental composite containing bismethylene spiroorthocarbonate expanding monomer. *Int. J. Mol. Sci.* **2014**, *15*, 2400–2412.
36. Berlanga Duarte ML, Reyna Medina LA, Torres Reyes P, Esparza González SC, Herrera González AM. Dental restorative composites containing methacrylic spiroorthocarbonate monomers as antishrinking matrixes. Journal of Applied Polymer Science. 2019 Mar 5;136(9):47114 https://doi.org/10.1002/app.47114
37. Podgórski M, Becka E, Claudino M, Flores A, Shah PK, Stansbury JW, Bowman CN. Ester-free thiol–ene dental restoratives—Part A: Resin development. *Dent. Mater.* **2015**. 31, 1255-1262.
38. He, J.; Garoushi, S.; Vallittu, P.K.; Lassila, L. Effect of low-shrinkage monomers on the physicochemical properties of experimental composite resin. *Acta Biomater. Odontol. Scand.* **2018**, *4*, 30–37. https://doi.org/10.1080/23337931.2018.1444488.
39. He, J.; Garoushi, S.; Säilynoja, E.; Vallittu, P.K.; Lassila, L. The effect of adding a new monomer "Phene" on the polymerization shrinkage reduction of a dental resin composite. *Dent. Mater.* **2019**, *35*, 627–635. https://doi.org/10.1016/j.dental.2019.02.006.
40. Herrera-González, A.M.; Pérez-Mondragón, A.A.; Cuevas-Suárez, C.E. Evaluation of bio-based monomers from isosorbide used in the formulation of dental composite resins. *J. Mech. Behav. Biomed. Mater.* **2019**, *100*, 103371. https://doi.org/10.1016/j.jmbbm.2019.103371.

41. González-López, J.A.; Pérez-Mondragón, A.A.; Cuevas-Suárez, C.E.; Trejo-Carbajal, N.; Herrera-González, A.M. Evaluation of dental composites resins formulated with non-toxic monomers derived from catechol. *J. Mech. Behav. Biomed. Mater.* **2020**, *104*, 103613. https://doi.org/10.1016/j.jmbbm.2019.103613.
42. González-López, J.A.; Pérez-Mondragón, A.A.; Cuevas-Suárez, C.E.; González, S.C.E.; Herrera-González, A.M. Dental composite resins with low polymerization stress based on a new allyl carbonate monomer. *J. Mech. Behav. Biomed. Mater.* **2020**, *110*, 103955.https://doi.org/10.1016/j.jmbbm.2020.103955.
43. Zhang, N.; Xie, C. Polymerization shrinkage, shrinkage stress, and mechanical evaluation of novel prototype dental composite resin. *Dent. Mater. J.* **2020**, *39*, 1064–1071. https://doi.org/10.4012/dmj.2019-286.
44. He, J.; Garoushi, S.; Säilynoja, E.; Vallittu, P.K.; Lassila, L. Physicochemical properties of dimethacrylate resin composites with comonomer of Hexa/Tri-ethylene glycol bis(carbamate-isopropyl-α-methylstyrene). *J. Mech. Behav. Biomed. Mater.* **2020**, *108*, 103832. https://doi.org/10.1016/j.jmbbm.2020.103832.
45. Bergeron, C.; Ballard, C.; Li, Y.; Zhang, W.; Zhong, Z.; Whang, K. A low-shrinkage, hydrophobic, degradation-resistant, antimicrobial dental composite using a fluorinated acrylate and an oxirane. *J. Appl. Biomater. Funct. Mater.* **2022**, *20*. https://doi.org/10.1177/22808000221087337.
46. de Lucena, F.S.; Logan, M.; Lewis, S.; Deatherage, N.; Furuse, A.Y.; Pfeifer, C.S. A novel low shrinkage dimethacrylate monomer as an alternative to BisGMA for adhesive and resin-based composite applications. *Braz. Oral Res.* **2024**, *38*, e097. https://doi.org/10.1590/1807-3107bor-2024.vol38.0097.
47. Luo, S.; Liu, F.; Yu, B.; He, J. Preparation of low shrinkage stress Bis-GMA free dental resin composites with a synthesized urethane dimethacrylate monomer. *J. Biomater. Sci. Polym. Ed.* **2019**, *30*, 137–149. https://doi.org/10.1080/09205063.2018.1556853.
48. Wang, X.; Huyang, G.; Palagummi, S.V.; Liu, X.; Skrtic, D.; Beauchamp, C.; Bowen, R.; Sun, J. High performance dental resin composites with hydrolytically stable monomers. *Dent. Mater.* **2018**, *34*, 228–237. https://doi.org/10.1016/j.dental.2017.10.007.
49. Filemban, H.; Bhadila, G.; Wang, X.; Melo, M.A.S.; Oates, T.W.; Weir, M.D.; Sun, J.; Xu, H.H. Novel low-shrinkage-stress bioactive nanocomposite with anti-biofilm and remineralization capabilities to inhibit caries. *J. Dent. Sci.* **2021**, *17*, 811–821. https://doi.org/10.1016/j.jds.2021.09.032.
50. Alhussein, A.; Alsahafi, R.; Wang, X.; Mitwalli, H.; Filemban, H.; Hack, G.D.; Oates, T.W.; Sun, J.; Weir, M.D.; Xu, H.H.K. Novel Dental Low-Shrinkage-Stress Composite with Antibacterial Dimethylaminododecyl Methacrylate Monomer. *J. Funct. Biomater.* **2023**, *14*, 335. https://doi.org/10.3390/jfb14070335.
51. Moraes, R.R.; Garcia, J.W.; Barros, M.D.; Lewis, S.H.; Pfeifer, C.S.; Liu, J.; Stansbury, J.W. Control of polymerization shrinkage and stress in nanogel-modified monomer and composite materials. *Dent. Mater.* **2011**, *27*, 509–519. https://doi.org/10.1016/j.dental.2011.01.006.
52. Song, H.B.; Sowan, N.; Shah, P.K.; Baranek, A.; Flores, A.; Stansbury, J.W.; Bowman, C.N. Reduced shrinkage stress via photo-initiated copper(I)-catalyzed cycloaddition polymerizations of azide-alkyne resins. *Dent. Mater.* **2016**, *32*, 1332–1342. https://doi.org/10.1016/j.dental.2016.07.014.
